# Establishing the link between microbial communities in bovine liver abscesses and the gastrointestinal tract

**DOI:** 10.1186/s42523-023-00278-0

**Published:** 2023-11-20

**Authors:** Lee J. Pinnell, J. Daniel Young, Tyler W. Thompson, Cory A. Wolfe, Tony C. Bryant, Mahesh N. Nair, John T. Richeson, Paul S. Morley

**Affiliations:** 1 Veterinary Education, Research, and Outreach Program, Texas A&M University, Canyon, TX 79015 USA; 2 Department of Agricultural Sciences, West Texas A&M University, Canyon, TX 79015 USA; 3 Department of Animal Sciences, Colorado State University, Fort Collins, CO 80523 USA; 4 Five Rivers Cattle Feeding, Johnstown, CO 80534 USA

**Keywords:** Liver abscesses, Gut health, Microbiome, Gut-liver axis

## Abstract

**Background:**

Liver abscesses (LAs) are one of the most common and important problems faced by the beef industry. The most efficacious method for the prevention of LAs in North America is through dietary inclusion of low doses of antimicrobial drugs such as tylosin, but the mechanisms by which this treatment prevents LAs are not fully understood. LAs are believed to result from mucosal barrier dysfunction in the gastrointestinal tract (GIT) allowing bacterial translocation to the liver via the portal vein, yet differences in the GIT microbiome of cattle with and without LAs have not been explored. Here, we characterized microbial communities from LAs, rumen, ileum, and colon from the same cattle for the first time.

**Results:**

Results demonstrate that tylosin supplementation was associated with differences in microbial community structure in the rumen and small intestine, largely because of differences in the predominance of Clostridia. Importantly, we show for the first time that microbial communities from multiple LAs in one animal’s liver are highly similar, suggesting that abscesses found at different locations in the liver may originate from a localized source in the GIT (rather than disparate locations). A large portion of abscesses were dominated by microbial taxa that were most abundant in the hindgut. Further, we identified taxa throughout the GIT that were differentially abundant between animals with and without liver abscesses. *Bifidobacterium* spp.—a bacteria commonly associated with a healthy GIT in several species—were more abundant in the rumen and ileum of animals without LAs compared to those with LAs.

**Conclusions:**

Together these results provide the first direct comparison of GIT and LA microbial communities within the same animal, add considerable evidence to the hypothesis that some LA microbial communities arise from the hindgut, and suggest that barrier dysfunction throughout the GIT may be the underlying cause of LA formation in cattle.

**Supplementary Information:**

The online version contains supplementary material available at 10.1186/s42523-023-00278-0.

## Background

Liver abscesses (LAs) are one of the most important and costly problems faced by the beef industry. In addition to condemned products, LAs can cause significant losses in the feedlot. Severe abscessation can reduce carcass yield and grade [1, 2], and result in reduced feed efficiency [3]. The most efficacious method for the prevention of LAs is through the inclusion of low doses of antimicrobial drugs (AMDs) in diets [3], and is used for this purpose in over 70% of feedlots with > 1000 animal capacity in the United States [4]. Randomized controlled trials have demonstrated that the in-feed supplementation of tylosin is associated with as much as a 50% reduction in LAs [5, 6]. Despite the efficacy of this prevention strategy, the prevalence of abscesses appears to be increasing. The US National Beef Quality Audits demonstrated that abscess prevalence has increased from 13.7% in 2011 to 20.7% in 2016 [7], and a similar increase from 13.3% (1999) to 22.0% (2016) in LA prevalence was identified in the Canadian Beef Quality Audits [8].

While tylosin is effective in reducing the prevalence of abscesses, the mechanisms by which it prevents LAs are not fully understood. All sequencing-based studies to date have concluded that there is no change in the diversity or composition of LA microbial communities resulting from in-feed supplementation of tylosin [9–13]. Antimicrobial drug exposures have been shown to impact microbial community structure of the gastrointestinal tract (GIT) in a variety of species, but there have been relatively few studies on the impacts of dietary tylosin supplementation on the composition of microbial communities in the GIT of cattle. These have not identified differences in microbial community structures in the rumen, cecum, colon, [14], nor in the feces of feedlot cattle [15, 16]. Antibiotic alternatives are currently being investigated to aid in the reduction and prevention of LAs due to growing concerns about antimicrobial resistance (AMR). Elucidating the mechanism by which tylosin impacts the microbial communities of the GIT and prevents LA occurrence should aid the ability to develop novel, efficacious prevention methods.

Historically, the accepted pathogenesis of liver abscesses has been attributed to a gut barrier dysfunction (GBD) in the rumen caused by acidosis. Extended feeding diets with high concentrations of fermentable starches are often thought to be the principal cause of this metabolic disorder and subsequent translocation of bacteria to the liver [3]. *Fusobacteria necrophorum* is widely considered the most common causative agent of LAs given its ubiquitous presence in culture-based investigations of LAs in cattle [2, 17–19]. Recently, culture-independent, sequencing-based investigations of LAs in cattle have all demonstrated that LAs are polymicrobial with members of five phyla (Fusobacteriota, Bacteroidiota, Proteobacteria, Firmicutes, and Actinobacteria) dominating > 99% of LA communities [9–13]. While *Fusobacterium* is still the most abundant community member when averaged across all LAs, the most recent investigations of microbial community structures of individual LAs have shown that a large proportion of LAs are actually dominated by members of Bacteroidetes (largely *Bacteroides* or *Porphyromonas*); microbial taxa more commonly associated with more distal portions of the GIT [13]. Yet, to date no study has investigated the microbial communities of LAs and multiple GIT locations within the same individual cattle.

This study utilized 16S rRNA gene sequencing to (1) investigate the impact of tylosin on microbial communities in LAs, and the rumen, ileum, and colon in the same animals, (2) compare microbial community structure among multiple LAs from one liver, (3) quantify prevalent LA taxa throughout the GIT, and (4) evaluate differences in microbial communities between abscessed and non-abscessed animals in the rumen, ileum, and colon. Importantly, it leverages samples collected as part of a randomized, controlled intervention trial and represents the first to investigate microbial diversity and composition in both multiple GIT locations and LAs of the same animals.

## Results

### Tylosin alters the diversity and composition of microbial communities within the bovine GIT but not liver abscesses

**Fig. 1:**
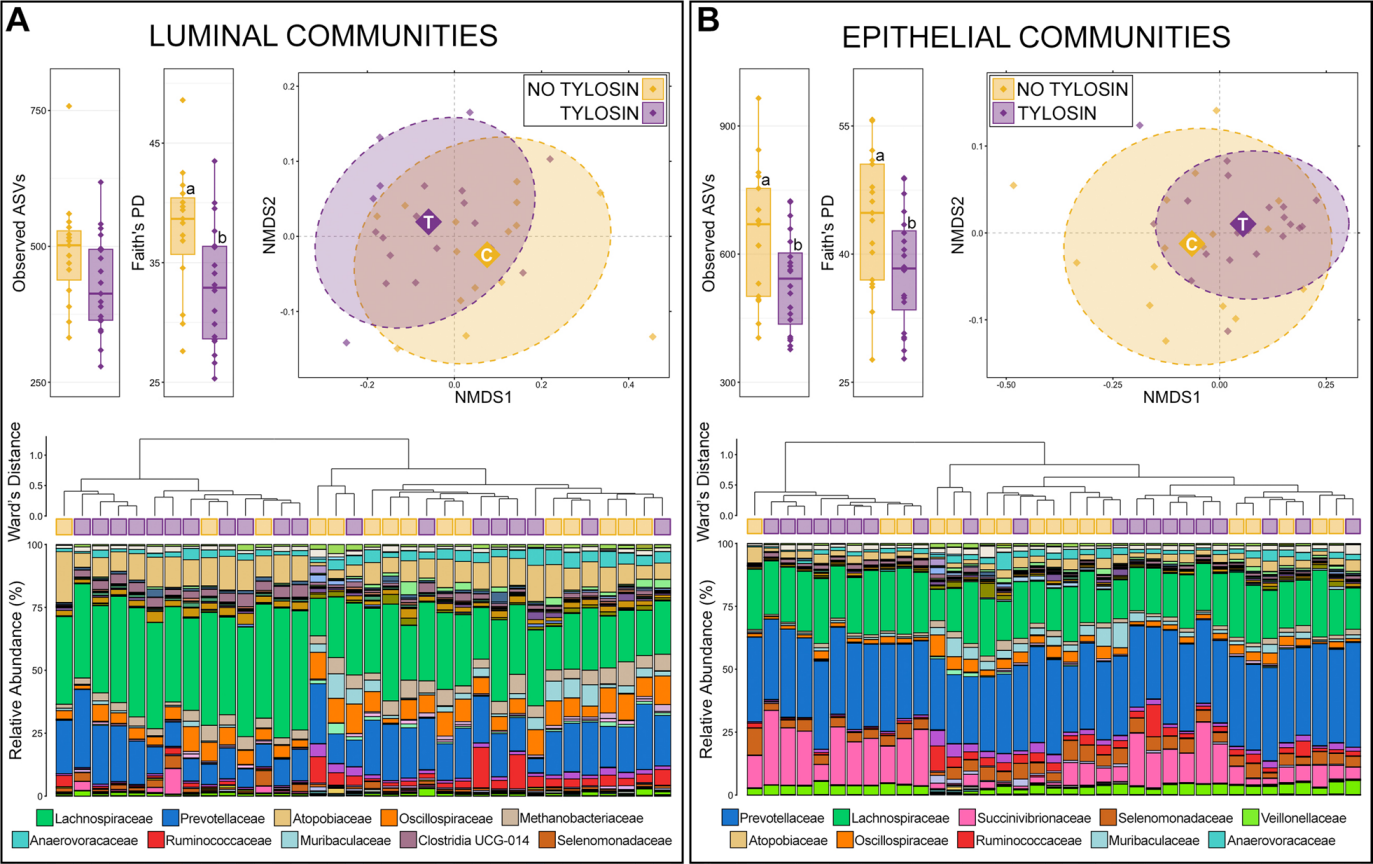
Alpha and beta-diversity in luminal (**A**) and epithelial (**B**) microbial communities of the rumen between animals that received tylosin supplementation and those that did not. Boxplots demonstrate differences in observed amplicon sequences variants (ASVs; richness) and Faith’s phylogenetic diversity. Significant differences in richness and diversity are noted by different letters (Kruskal–Wallis analysis of variance, *p* < 0.05, n = 15–20). Non-metric multidimensional scaling (NMDS) of generalized UniFrac distances illustrates differences in overall microbial community structure between treatment groups. The NMDS demonstrates clustering of 16S rRNA gene sequences from animals that received tylosin (purple) and those that did not (gold). The large opaque points represent the centroid for communities from each treatment group, while the smaller and more transparent points represent the individual animals within each group. Dashed lines and shaded areas represent 90% confidence intervals. Tylosin supplementation resulted in significant differences in the overall composition of luminal and epithelial communities in the rumen (PERMANOVA, *p* < 0.05, n = 15–20). Dendrogram displaying the relatedness of luminal and epithelial communities in the rumen based on normalized ASVs. Hierarchal clustering was performed on generalized UniFrac distances using Ward’s agglomeration method. Purple boxes represent communities from animals that received tylosin supplementation and gold boxes represent communities from animals that did not. The bar plot illustrates the relative abundance of microbial families with each individual sample. The 10 most abundant families are displayed in the legend

*Rumen microbial communities.* Both the richness and diversity of luminal and epithelial microbial communities in the rumen were lower in animals receiving tylosin supplementation, and except for richness in luminal communities (*p* = 0.07), the decreases were statistically significant (Fig. 1; Kruskal–Wallis, n = 15–20, *p* < 0.05). Based on generalized UniFrac distances, the overall composition of luminal and epithelial communities in the rumen were significantly different between animals that received tylosin supplementation and those that did not (Fig. 1; PERMANOVA, n = 15–20, *p* < 0.05).

Hierarchal clustering further revealed that there were two major clades of both luminal and epithelial rumen communities. The two clades of luminal communities were largely the result of differences in the relative abundance of Lachnospiraceae (Fig. 1). The clade with lower relative abundances of Lachnospiraceae (right side of dendrogram) was largely made up of communities from cattle that did not receive tylosin supplementation (12/15; 80.0% of cattle not receiving tylosin). While communities from cattle that received tylosin were more evenly split between the two clades, the majority (11/19; 57.9%) were in the clade with higher Lachnospiraceae abundances.

Across all luminal communities, 15 families comprised more than 1% of the overall community and together these 15 families represented 89.03% of the luminal community in the rumen. Lachnospiraceae was the most abundant family, followed Prevotellaceae, Atopobiaceae, Oscillospiraceae, Methanobacteriaceae, Anaerovoracaceae, Ruminococcaceae, Muribaculaceae, Clostridia UCG-014, Erysipelotrichaceae (Fig. 1; Additional file 1: Table S1). Of the 15 families comprising more than 1% of the overall community, Lachnospiraceae (27.14% ± 2.516—no tylosin; 35.39% ± 1.984—tylosin) and Clostridia UCG-014 (1.56% ± 0.238—no tylosin; 2.88% ± 0.313—tylosin) had significantly higher relative abundances in communities from animals that received tylosin compared to those that did not (Additional file 1: Table S1; Kruskal–Wallis analysis of variance; n = 15–19; *p* < 0.05).

Within rumen epithelial communities, the two major clades formed largely on differences in the relative abundance of Succinivibrionaceae and less abundant families (Fig. 1). The clade with lower relative abundance of Succinivibrionaceae (right side of dendrogram) contained nearly all the communities from cattle that did not receive tylosin supplementation (14/17; 82.4%; Fig. 1). Within that same clade however, communities from the few cattle that did receive tylosin mainly clustered together within a sub-clade marked by higher relative abundances of Succinivibrionaceae. Across all epithelial communities, 14 families comprised more than 1% of the overall community, and together these families represented 91.26% of the epithelial community in the rumen. Prevotellaceae was the most abundant family, followed by Lachnospiraceae, Succinivibrionaceae, Selenomonadaceae, Veillonellaceae, Atopobiaceae, Oscillospiraceae, Ruminococcaceae, Muribaculaceae, and Anaerovoracaceae (Additional file 1: Table S1). Of the 14 families comprising more than 1% of the community, Succinivibrionaceae (6.34% ± 1.252—no tylosin; 14.29% ± 1.798—tylosin), Veillonellaceae (2.85% ± 0.309—no tylosin; 3.64% ± 0.241—tylosin), and Clostridia UCG-014 (0.79% ± 0.094—no tylosin; 1.23% ± 0.122—tylosin) were significantly more abundant in animals that received tylosin, while Oscillospiraceae (3.43% ± 0.430—no tylosin; 2.22% ± 0.232—tylosin), Ruminococcaceae (3.01% ± 0.514 – no tylosin; 2.33% ± 0.693 – tylosin), and Rikenellaceae (2.33% ± 0.393—no tylosin; 1.31% ± 0.177—tylosin) were less abundant in animals that received tylosin compared to those that did not (Additional file 1: Table S1; Kruskal–Wallis analysis of variance; n = 17–20, *p* < 0.05).

**Fig. 2:**
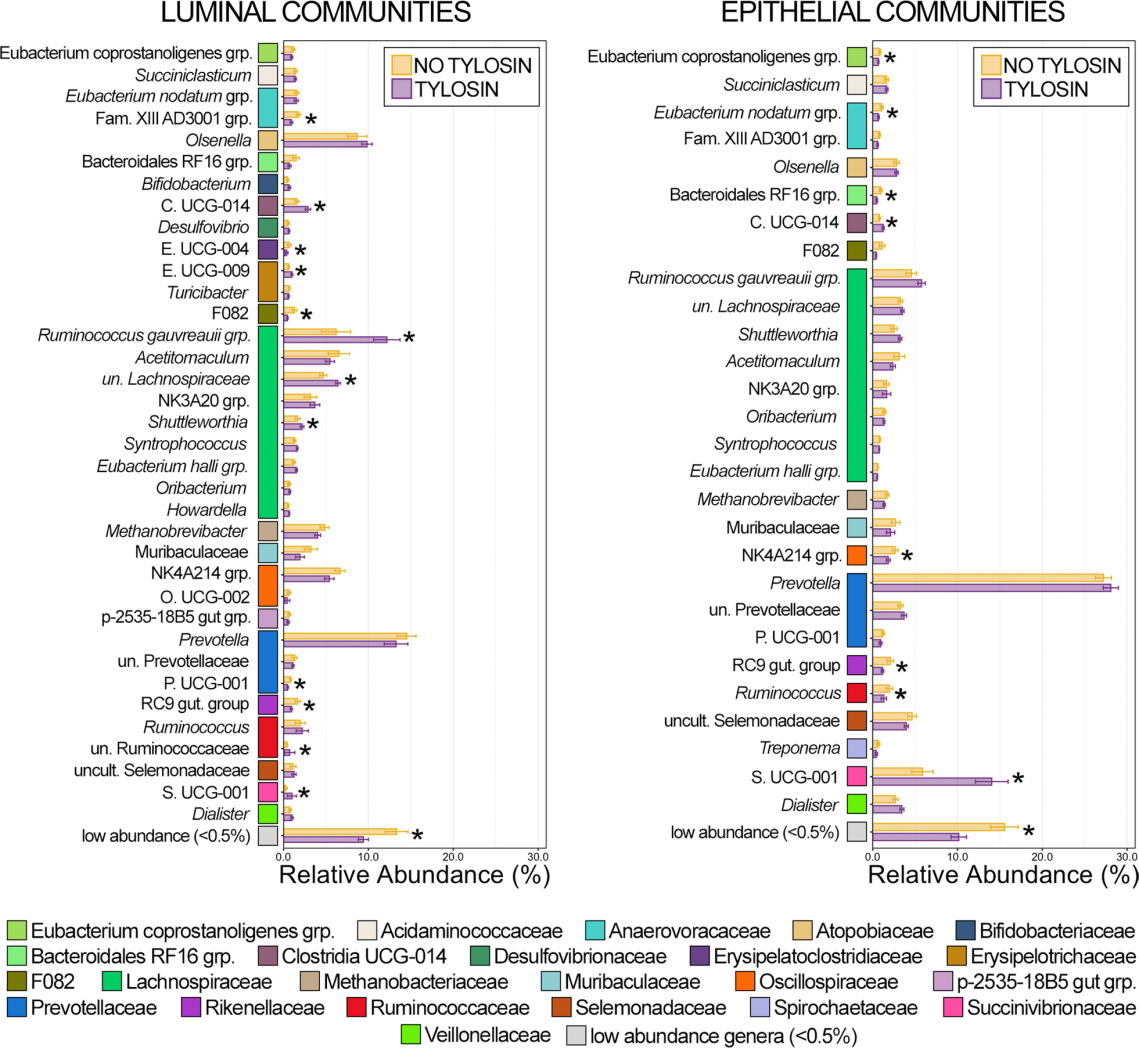
Bar plots demonstrating the mean relative abundance of all genera within luminal and epithelial communities of the rumen that comprised at least 0.5% of the overall microbial community between animals that received tylosin supplementation and those that did not. Colored boxes represent the family that the genus belongs to and correspond to the colors representing the family in Fig. 1. All families are listed in the legend. Error bars represent the standard error of the mean. Significant differences between animals that received tylosin and those that did not are noted by an asterisk (Kruskal–Wallis analysis of variance; n = 15–20; *p* < 0.05)

Of the 36 genera comprising at least 0.5% of the overall rumen luminal community, 12 were differentially abundant between animals that received tylosin and those that did not, while 9 of 28 genera comprising at least 0.5% of the overall epithelial community were differentially abundant (Fig. 2; Kruskal–Wallis analysis of variance, n = 15–20, *p* < 0.05). With the exception of *Ruminococcus*, all genera exhibited the same response to tylosin supplementation (i.e., increased or decreased predominance) in both luminal and epithelial rumen communities (Fig. 2). Clostridia UCG-014 and Succinivibrionaceae UCG-001 were in significantly greater relative abundance in both luminal and epithelial communities from the rumen of animals receiving tylosin compared to those that did not (Fig. 2; Kruskal–Wallis analysis of variance; n = 15–20, *p* < 0.05). Erysipelotrichaceae UCG-009 was more abundant within luminal rumen communities from tylosin supplemented animals but was below 0.5% relative abundance in epithelial communities. Members of the Ruminococcus gauvreauii group and *Shuttleworthia* were more abundant within luminal and epithelial communities from the rumen of animals receiving tylosin supplementation, but the difference was only significant in luminal communities (Fig. 2; Kruskal–Wallis analysis of variance; n = 15–20, *p* < 0.05). Only members of Rikenellaceae RC9 gut group had significantly lower relative abundances in both luminal and epithelial communities. While members of Eubacterium coprostanoligenes group, Eubacterium nodatum group, Family XIII AD3001 group, Bacteroidales RF16 group, F082, NK4A214 group, and Prevotellaceae UCG-001 all decreased, the difference was only statistically significant in either luminal or epithelial communities (Fig. 2; Kruskal–Wallis analysis of variance, n = 15–20, *p* < 0.05). In both luminal and epithelial rumen communities, low abundance genera (< 0.5% RA) were significantly less abundant collectively in animals that received tylosin supplementation. Interestingly, most of the differentially abundant genera across all rumen communities were all members of the same class; with 10 of the 18 genera being members of the class Clostridia (Fig. 2, Additional file 2: Table S2).

#### Ileum microbial communities

**Fig. 3:**
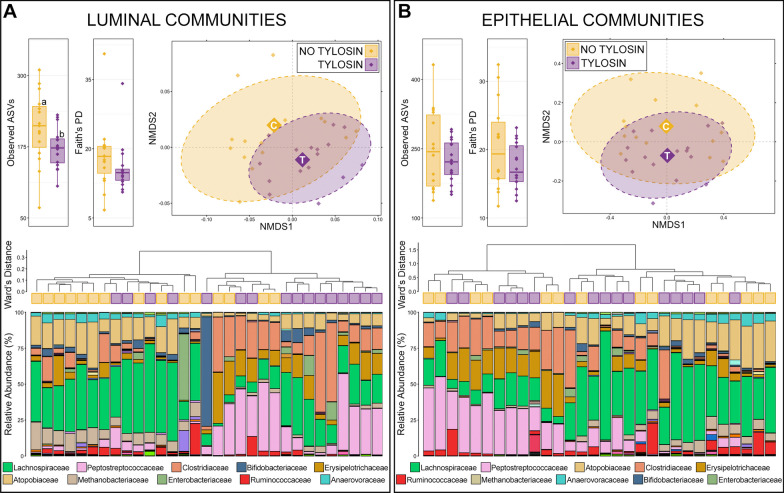
Alpha and beta-diversity in luminal and epithelial microbial communities of the small intestine between animals that received tylosin supplementation and those that did not. Boxplots demonstrate differences in observed amplicon sequences variants (ASVs; richness) and Faith’s phylogenetic diversity. Luminal communities from the ileum of animals receiving tylosin were significantly less rich that those that did not (Kruskal–Wallis analysis of variance, *p* < 0.05, n = 15–19). Non-metric multidimensional scaling (NMDS) of generalized UniFrac distances illustrates differences in overall microbial community structure between treatment groups. The NMDS demonstrates clustering of 16S rRNA gene sequences from animals that received tylosin (purple) and those that did not (gold). The large opaque points represent the centroid for communities from each treatment group, while the smaller and more transparent points represent the individual animals within each group. Dashed lines and shaded areas represent 90% confidence intervals. Tylosin supplementation resulted in significant differences in the overall composition of luminal communites but not epithelial communities of the small intestine (PERMANOVA, *p* > 0.05, n = 15–19). Dendrogram displaying the relatedness of luminal and epithelial communities in the large intestine based on normalized ASVs. Hierarchal clustering was performed on generalized UniFrac distances using Ward’s agglomeration method. Purple boxes represent communities from animals that received tylosin supplementation and gold boxes represent communities from animals that did not. The bar plot illustrates the relative abundance of microbial families with each individual sample. The 10 most abundant families are displayed in the legend

Animals that received tylosin had significantly lower richness in ileum microbial communities found in the lumen, but not on the epithelium (Fig. 3; Kruskal–Wallis analysis of variance, n = 15–16, *p* < 0.05). While diversity was lower in both luminal and epithelial communities from the ileum of animals that received tylosin, this difference was not statistically significant. Based on generalized UniFrac distances, the composition of luminal communities in the ileum were significantly different between animals given diet supplemented with tylosin and those not receiving tylosin (Fig. 3; PERMANOVA, n = 15–16, *p* < 0.05). Despite the lack of statistical significance (*p* = 0.09) the amount of variation in community composition explained by tylosin supplementation in the epithelium of the ileum (*R*^2^ = 0.06) was similar to statistically significant differences in the rumen and lumen of the ileum (Additional file 1: Table S1).

Hierarchal clustering further revealed that two clades containing nearly equal numbers of communities formed for luminal communities in the ileum (Fig. 3). The clade on the left had elevated relative abundances of Lachnospiraceae, Atopobiaceae, Anaerovoraceae, and Ruminococcaceae, and 11/15 (73.3%) communities from animals that did not receive tylosin were within the clade. The right clade had higher abundances of Peptostreptococcaceae, Erysipelotrichaceae, and Clostridiaceae, and 12/16 communities from animals that received tylosin supplementation were within the clade (Fig. 3). Across all luminal communities in the ileum, 10 families comprised more than 1% of the community, and together these families represented 95.4% of the overall community. Lachnospiraceae was the most abundant, followed by Peptostreptococcaceae, Clostridiaceae, Atopobiaceae, Erysipelotrichaceae, Methanobacteriaceae, Bifidobacteriaceae, Enterobacteriaceae, Rumincoccaceae, and Anaerovoracaceae (Additional file 3: Table S3). Of the 10 families comprising more than 1% of ileum luminal communities, Methanobacteriaceae (7.76% ± 1.295—no tylosin; 2.96% ± 0.601—tylosin), Ruminococcaceae (3.74% ± 1.348—no tylosin; 1.74% ± 0.838—tylosin) and Anaerovoraceae (3.55% ± 0.600—no tylosin; 1.18% ± 0.276—tylosin) were significantly less abundant in communities from animals that received tylosin compared to those that did not (Additional file 3: Table S3; Kruskal–Wallis analysis of variance, n = 15–16, *p* < 0.05).

In ileum epithelial communities, hierarchal clustering revealed that the two major clades formed based on differing relative abundances of Lachnospiraceae, Peptostreptococcaceae, Clostridiaceae, Atopobiaceae, Erysipelotrichaceae, and Anaerovoracaceae as was the case in luminal communities (Fig. 3). However, communities from animals that received tylosin and animals that did not were more interspersed across these two major clades. Across all epithelial communities in the ileum, 9 families comprised more than 1% of the community, and together these families represented 94.1% of the overall community. Lachnospiraceae was the most abundant, followed by Peptostreptococcaceae, Atopobiaceae, Clostridiaceae, Erysipelotrichaceae, Rumincoccaceae, Methanobacteriaceae, Anaerovoracaceae, and Bifidobacteriaceae (Additional file 3: Table S3). Of these 9 families, Ruminococcaceae (5.99% ± 1.504—no tylosin; 2.61% ± 1.074—tylosin) was significantly less abundant in communities from animals that received tylosin compared to those that did not (Additional file 3: Table S3; Kruskal–Wallis analysis of variance, n = 15–19, *p* < 0.05).

**Fig. 4:**
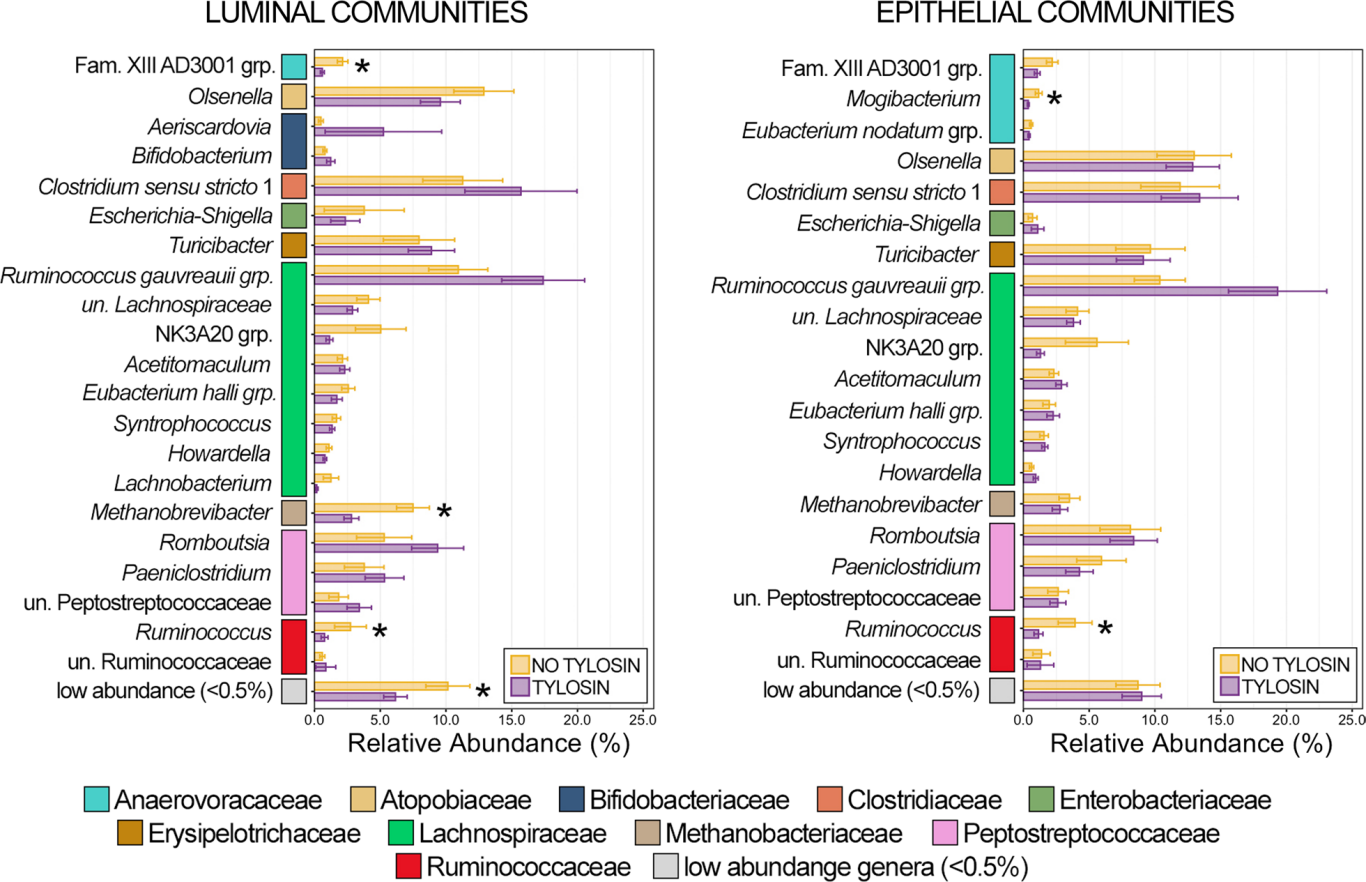
Bar plots demonstrating the mean relative abundance of all genera within luminal and epithelial communities of the small intestine that comprised at least 0.5% of the overall microbial community between animals that received tylosin supplementation and those that did not. Colored boxes represent the family that the genus belongs to and correspond to the colors representing the family in Fig. 3. All families are listed in the legend. Error bars represent the standard error of the mean. Significant differences between animals that received tylosin and those that did not are noted by an asterisk (Kruskal–Wallis analysis of variance; n = 15–20; *p* < 0.05)

There were 21 genera that comprised more than 0.5% of the luminal community in the ileum of which 3 were differentially abundant, and of the 20 genera that comprised more than 0.5% of the epithelial community, two were differentially abundant (Fig. 4; Kruskal–Wallis analysis of variance, n = 15–19, *p* < 0.05). In general, there was considerably greater animal to animal variation in community structure in ileum communities compared to the rumen and large intestine. As a result of this variation, there were no genera with statistically higher relative abundance in luminal communities from animals that received tylosin, though some of the most abundant genera (i.e., Ruminococcus gauvreauii group, *Clostridium senso stricto 1*, *Romboutsia*) all exhibited a trend of increase relative abundance (Fig. 4). Similary, the Ruminococcus gauvreauii group was the most abundant genus within epithelial communities, and while the genus’ abundance was higher in animals that received tylosin it was not statistically significant (*p* = 0.07). *Ruminococcus* was in significantly lower relative abundance in cattle that received tylosin in both luminal and epithelial communities, while relative abundances of Family XIII AD3001 group and *Methanobrevibacter* were significantly lower in just luminal communities, and *Mogibacterium* was significantly lower in epithelial communities only (Fig. 4; Kruskal–Wallis analysis of variance, n = 15–19, *p* < 0.05). Collectively, low abundance genera (< 0.5% RA) had significantly lower relative abundance within animals that received tylosin. The majority of differentially abundant genera in the ileum between animals that received tylosin and those that did not belonged to the class Clostridia (3/4; 75%; Additional file 2: Table S2).

#### Colon microbial communities

**Fig. 5:**
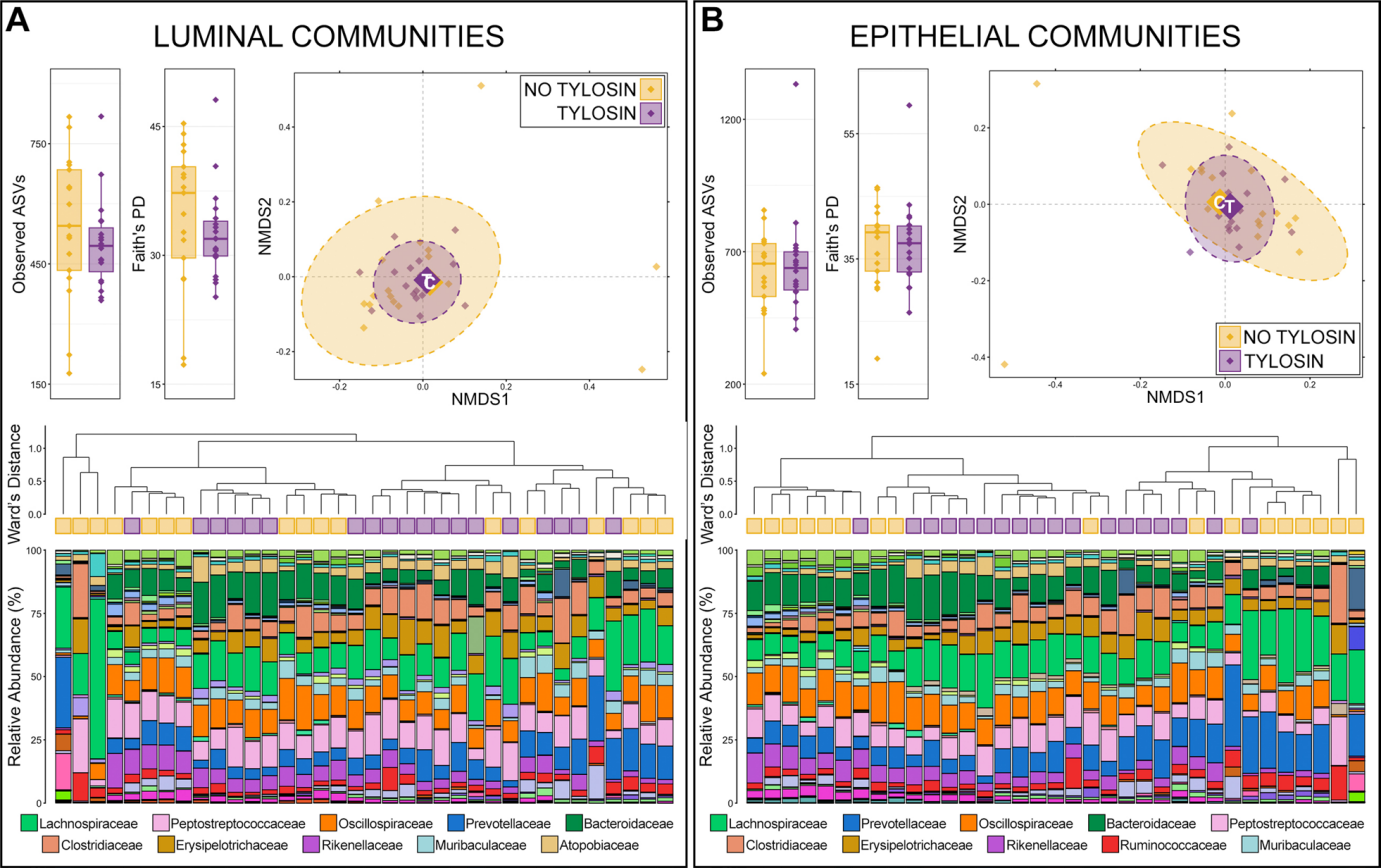
Alpha and beta-diversity in luminal and epithelial microbial communities of the large intestine between animals that received tylosin supplementation and those that did not. Boxplots demonstrate differences in observed amplicon sequences variants (ASVs; richness) and Faith’s phylogenetic diversity. Significant differences in richness and diversity are noted by different letters (Kruskal–Wallis analysis of variance, *p* < 0.05, n = 15–19). Non-metric multidimensional scaling (NMDS) of generalized UniFrac distances illustrates differences in overall microbial community structure between treatment groups. The NMDS demonstrates clustering of 16S rRNA gene sequences from animals that received tylosin (purple) and those that did not (gold). The large opaque points represent the centroid for communities from each treatment group, while the smaller and more transparent points represent the individual animals within each group. Dashed lines and shaded areas represent 90% confidence intervals. Tylosin supplementation resulted in significant differences in the overall composition of luminal communities but not epithelial communities in the small intestine (PERMANOVA, *p* < 0.05, n = 15–19). Dendrogram displaying the relatedness of luminal and epithelial communities in the small intestine based on normalized ASVs. Hierarchal clustering was performed on generalized UniFrac distances using Ward’s agglomeration method. Purple boxes represent communities from animals that received tylosin supplementation and gold boxes represent communities from animals that did not. The bar plot illustrates the relative abundance of microbial families with each individual sample. The 10 most abundant families are displayed in the legend

There were no significant differences in the richness or diversity of luminal or epithelial communities in the colon of animals that received tylosin compared to those that did not (Fig. 5; Kruskal–Wallis analysis of variance, n = 17–19, *p* > 0.05). Based on generalized UniFrac distances, there was no significant difference in overall community structure in colon luminal or epithelial communities from animals that received tylosin compared to those that did not (Fig. 5; PERMANOVA, n = 17–19). Hierarchal clustering further revealed that, with the exception of 2–3 outlier animals, there were 2 major clades of luminal communities and 3 major clades of epithelial communities in the colon (Fig. 5). At the family level, the two luminal clades were mainly separated by differing relative abundances of Bacteroidaceae, and communities from animals that received tylosin and those that did not are interspersed between the two major clades (Fig. 5). However, within each of the major clades, communities tended to form smaller sub-clades with like samples (i.e., tylosin supplemented communities with other tylosin supplemented communities). Across all luminal communities, there were 18 families that comprised more than 1% of the community, and together these families represented 91.9% of the overall community. Lachnospiraceae was the most abundant family, followed by Peptostreptococcaceae, Oscillospiraceae, Prevotellaceae, Bacteroidaceae, Clostridiaceae, Eryspilotrichaceae, Rikenellaceae, Muribaculaceae, and Atopobiaceae (Additional file 4: Table S4). Of the 18 families comprising more than 1% of the luminal community, Eryspilotrichaceae (4.91% ± 0.823—no tylosin; 7.97% ± 0.619—tylosin), Atopobiaceae (2.33% ± 0.249—no tylosin; 4.22% ± 0.547—tylosin), and Methanobacteriaceae (1.69% ± 0.411—no tylosin; 2.22% ± 0.267—tylosin) were more abundant in large intestine luminal communities from animals that received tylosin compared to those that did not, while Anaerovoracaceae (1.68% ± 0.476—no tylosin; 1.01% ± 0.084—tylosin) was less abundant (Additional file 4: Table S4; Kruskal–Wallis analysis of variance, n = 17–19, *p* < 0.05).

Within epithelial communities in the colon, the major clades also formed largely as a result of differing relative abundances of Bacteroidaceae (Fig. 5). Communities from animals that received tylosin and those that did not were again interspersed between major clades, but formed sub-clades that were largely comprised of only like samples (Fig. 5). Across all epithelial communities, 18 families comprised more than 1% of the community, and together these families represented 89.8% of the overall community. Lachnospiraceae was the most abundant family, followed by Prevotellaceae, Oscillospiraceae, Bacteroidaceae, Peptostreptococcaceae, Clostridiaceae, Eryspilotrichaceae, Rikenellaceae, Ruminococcaceae, and Muribaculaceae (Additional file 4: Table S4). Of the 18 families comprising more than 1% of the community, Eryspilotrichaceae (4.40% ± 0.611 – no tylosin; 6.47% ± 0.544 – tylosin), Atopobiaceae (1.94% ± 0.203 – no tylosin; 3.59% ± 0.490 – tylosin), and Methanobacteriaceae (0.90% ± 0.239 – no tylosin; 1.28% ± 0.172 – tylosin) were in significantly higher relative abundance in animals that received tylosin compared to those that did not (Additional file 4: Table S4; Kruskal–Wallis analysis of variance, n = 17–18, *p* < 0.05).

**Fig. 6:**
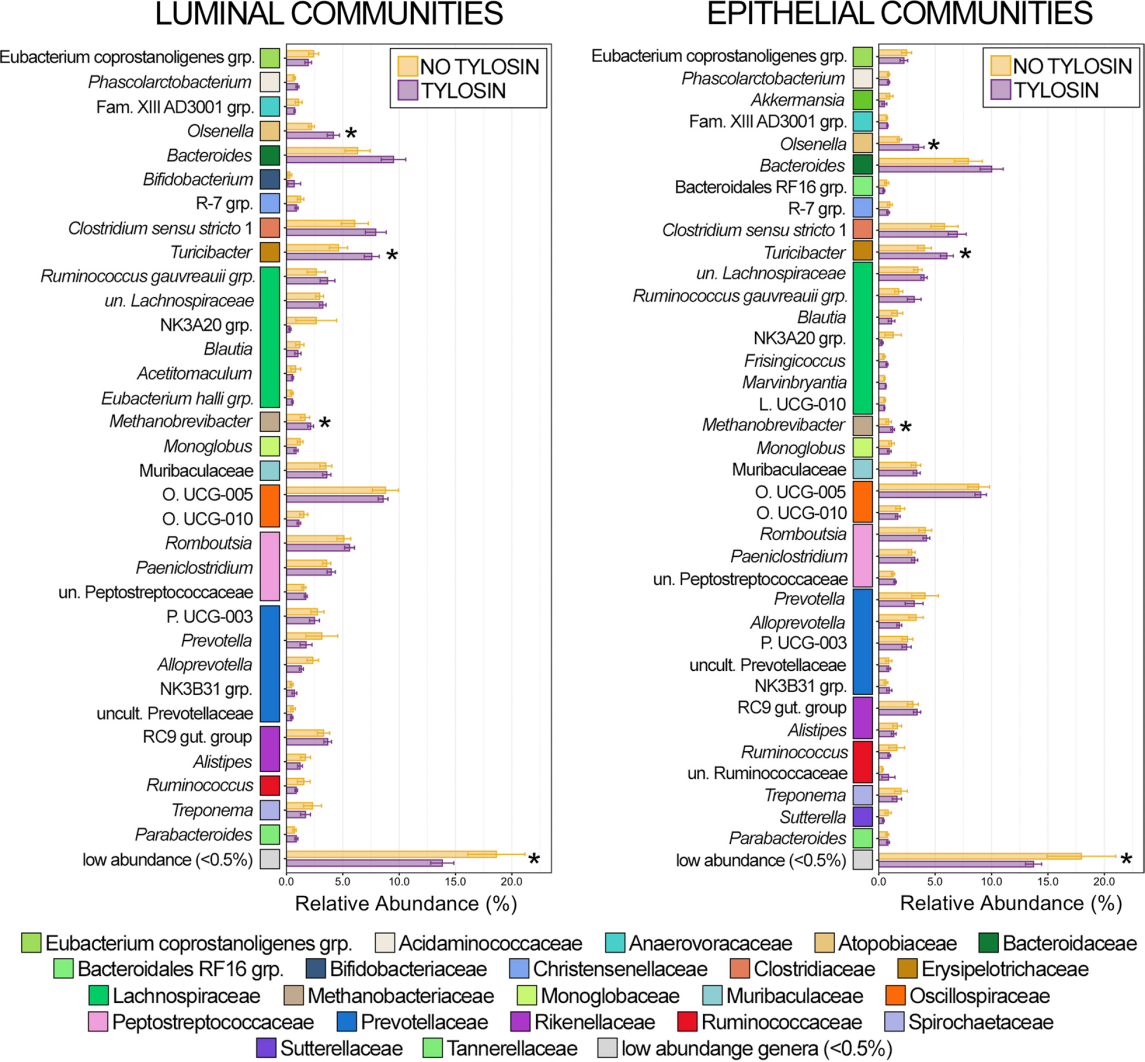
Bar plots demonstrating the mean relative abundance of all genera within luminal and epithelial communities of the large intestine that comprised at least 0.5% of the overall microbial community between animals that received tylosin supplementation and those that did not. Colored boxes represent the family that the genus belongs to and correspond to the colors representing the family in Fig. 1. All families are listed in the legend. Error bars represent the standard error of the mean. Significant differences between animals that received tylosin and those that did not are noted by an asterisk (Kruskal–Wallis analysis of variance; n = 15–20; *p* < 0.05)

Of the 33 genera that comprised more than 0.5% of the overall luminal community three were differentially abundant between animals that received tylosin and those that did not, while three of the 37 genera that comprised more than 0.5% of epithelial communities were differentially abundant. *Olsenella, Turicibacter*, and *Methanobrevibacter* were in significantly higher relative abundance in luminal and epithelial communities from animals that received tylosin (Fig. 6; Kruskal–Wallis analysis of variance, n = 17–19, *p* < 0.05). Similar to the rumen and ileum, low abundance genera (< 0.5% RA) were once again collectively less abundant in luminal and epithelial communities from the colon of animals that received tylosin (Fig. 6; Kruskal–Wallis analysis of variance, n = 17–19, *p* < 0.05). Unlike in the rumen and small intestine however, none of the differentially abundant genera in the large intestine belonged to Clostridia (Fig. 6, Additional file 2: Table S2).

#### Liver abscess communities

Based on generalized UniFrac distances, there were no significant differences in community composition within LA purulent material from animals that received tylosin compared to those that did not (Additional file 5: Figure S1; PERMANOVA, n = 10, *p* > 0.05). Six genera (*Fusobacterium, Bacteroides, Trueperella, Porphyromonas, Parvimonas,* and *Helcococcus*) each comprised more than 0.5% of the overall community, and collectively they represented 97.5% of the liver abscess community. None of these six genera were differentially abundant between animals that received tylosin and those that did not (Additional file 5: Figure S1; Kruskal–Wallis analysis of variance, n = 10, *p* > 0.05).

### Multiple liver abscesses from the same animal have similar microbial communities

**Fig. 7:**
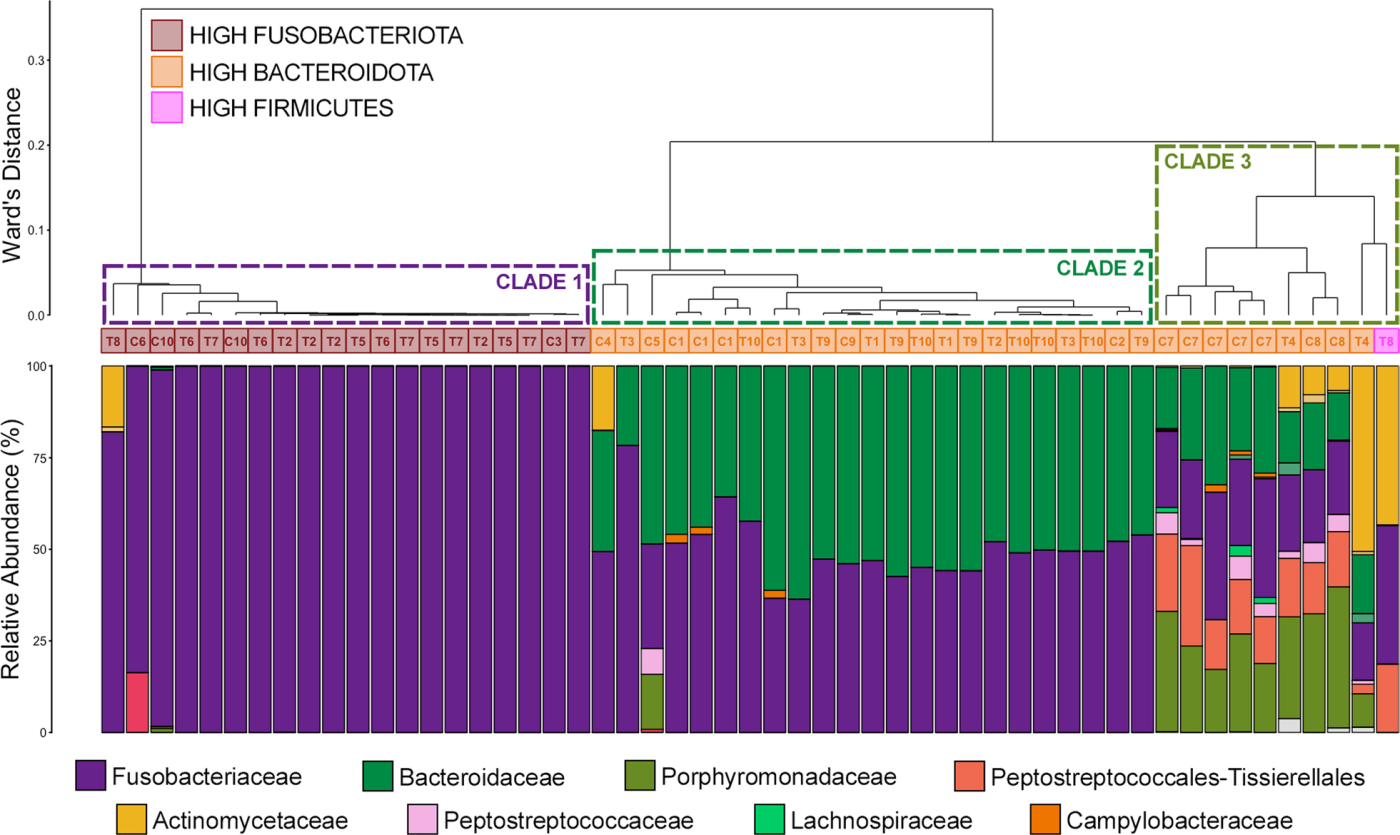
Hierarchal clustering using Ward’s agglomeration method on all liver abscess communities (n = 53) collected from 20 animals with liver abscesses illustrating the relatedness of communities classified as high Fusobacteria (dark red boxes), high Bacteroidetes (orange boxes), or high Firmicutes (magenta boxes) based on the parameters set in Pinnell et al., 2022. These community types are further classified into three clades with markedly different community structure; a Fusobacteriaceae-dominated clade (clade 1, purple dashed outline), a high Bacteroidaceae clade (clade 2, sea green dashed outline), and high diversity clade comprised primarly of Bacteroidaceae, Porphyromonas, Peptostreptococcales-Tissierellales, Actinomycetaceae, and Peptostreptococcaceae (clade 3, olive green dashed outline). The text within the boxes represents the animals the abscess was collected from, with ‘T’ signifying an animal that received tylosin supplementation and a ‘C’ signifying an animal that did not. The bar plot illustrates the relative abundance of microbial families with each individual sample. The 8 most abundant families are displayed in the legend

Of the 20 animals with liver abscesses that were sampled, 14 had multiple abscesses (Fig. 7A). Of the 53 total abscesses sampled, 47 of them were collected from animals with multiple abscesses and 6 were collected from animals with only one abscess. The composition of microbial communities of abscesses collected from the same liver were remarkably similar (Additional file 6: Figure S2). Based on the parameters used by Pinnell et al. [9] (e.g., high Fusobacteria =  > 75% Fusobacteria RA; high Bacteroidetes =  > 14% Bacteroidetes RA; high Firmicutes =  > 11% Firmicutes RA) multiple LAs from the same liver would all be classified as the same community type, except for two animals (ID# T2 had 3 high Fusobacteria and 1 high Bacteroidetes abscesses and ID# T8 had 1 high Fusobacteria and 1 abscess classified as “other” in community structure). Despite being classified as different community types, the two communities from one steer-ID# T8—were similar and exhibited high relative abundances of Fusobacteriaceae and Actinomycetaceae and the different community type classification was solely the result of Fusobacteriaceae being above or below the threshold of 75% relative abundance (Fig. 7; Additional file 5: Figure S2). Further, except for steer-ID# T2 and steer-ID# T8, all liver abscess communities from the same animal fell within the same major clade, of which there were three across all liver abscesses (Fig. 7). Clade 1 contained communities from abscesses dominated by Fusobacteriaceae, clade 2 contained abscesses with higher relative abundances of Bacteroidaceae, and clade 3 contained abscesses with more diversity, largely a result of elevated relative abundances of Porphyromonadaceae, Peptostreptococcales-Tissierellales, Peptostreptococcaceae, and in some cases Actinomycetaceae (Fig. 7).

### Linking liver abscesses microbial communities to GIT communities

#### Prevalent liver abscess taxa within GIT communities of the same individuals

**Fig. 8:**
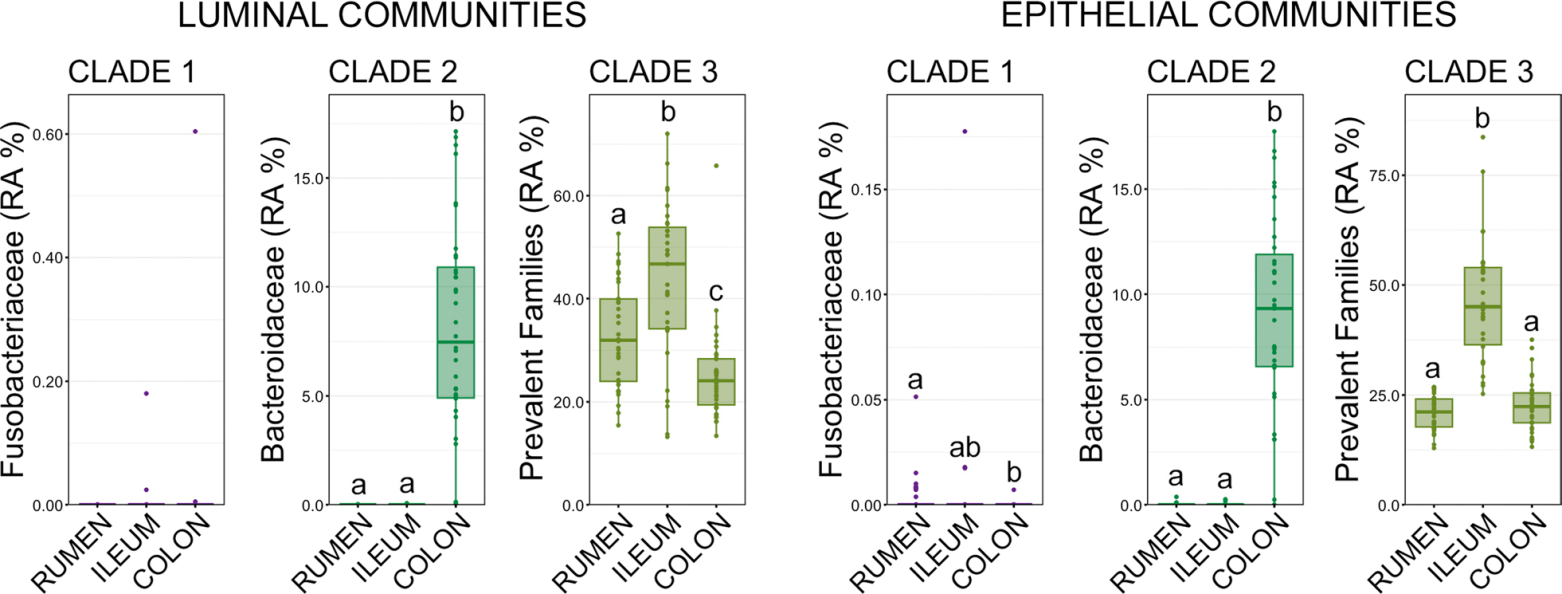
Boxplots demonstrating the relative abundances of prevalent families within each of the three clades of liver abscesses in luminal and epithelial communities of the gut. Significant differences between GIT locations are indicated by different letters (pairwise Wilcoxon rank-sum, n = 15–20, *p* < 0.05)

To investigate potential sources of liver abscesses communities, the prevalent families within each of the three liver abscesses community types (i.e., clade 1, clade 2, clade 3) were quantified in luminal and epithelial communities of the rumen, small intestine, and large intestine. Fusobacteriaceae was the only prevalent family within clade 1 abscesses and Bacteroidaceae was the only family within clade 2 abscesses. Porphyromonadaceae, Peptostreptococcales-Tissierellales, Peptostreptococcaceae, Actinomycetaceae, and Atopobiaceae were the prevalenet families within clade 3. There were no significant differences in the abundance of Fusobacteriaceae in luminal communities within the rumen, small intestine, or large intestine (Fig. 8; pairwise Wilcoxon rank-sum analysis of variance, n = 15–20, *p* > 0.05). However, Fusobacteriaceae were in significantly higher relative abundance within rumen epithelium communities as compared to colon epithelium communities (Fig. 8; pairwise Wilcoxon rank-sum analysis of variance, n = 15–20, *p* > 0.05). Given the sparsity of detection and extremely low relative abundance (< 0.05% in all but 2 communities) of Fusobacteriaceae the biological significance of this finding should be interpreted with caution. Bacteroidaceae was in significantly higher relative abundance within luminal and epithelial communities in the large intestine compared to both the rumen and small intestine (Fig. 8; pairwise Wilcoxon rank-sum analysis of variance, n = 15–20, *p* < 0.05). Bacteroidaceae was almost entirely comprised of *Bacteroides*, and unsurprisingly that genus was also significantly more abundant in the large intestine (Additional file 7: Figure S3; pairwise Wilcoxon rank-sum analysis of variance, n = 15–20, *p* < 0.05). In fact, *Bacteroides* was nearly absent across all rumen (0.011% ± 0.006 SEM) and small intestine communities (0.010% ± 0.005 SEM), while comprising 8.49% ± 0.572 SEM of the overall community in the large intestine. Prevalent families within clade 3 abscess communities were in significantly higher relative abundances within small intestine epithelial communities than rumen and large intestine epithelial communities (Fig. 8; pairwise Wilcoxon rank-sum analysis of variance, n = 15–20, *p* < 0.05). Within luminal communities, clade 3 prevalent families were more abundant in the small intestine than both the rumen and colon, and also significantly more abundant in the rumen as compared to large intestine luminal communities (Fig. 8; pairwise Wilcoxon rank-sum analysis of variance, n = 15–20, *p* < 0.05).

#### Differences in bovine GIT community composition between animals with and without liver abscesses

**Fig. 9:**
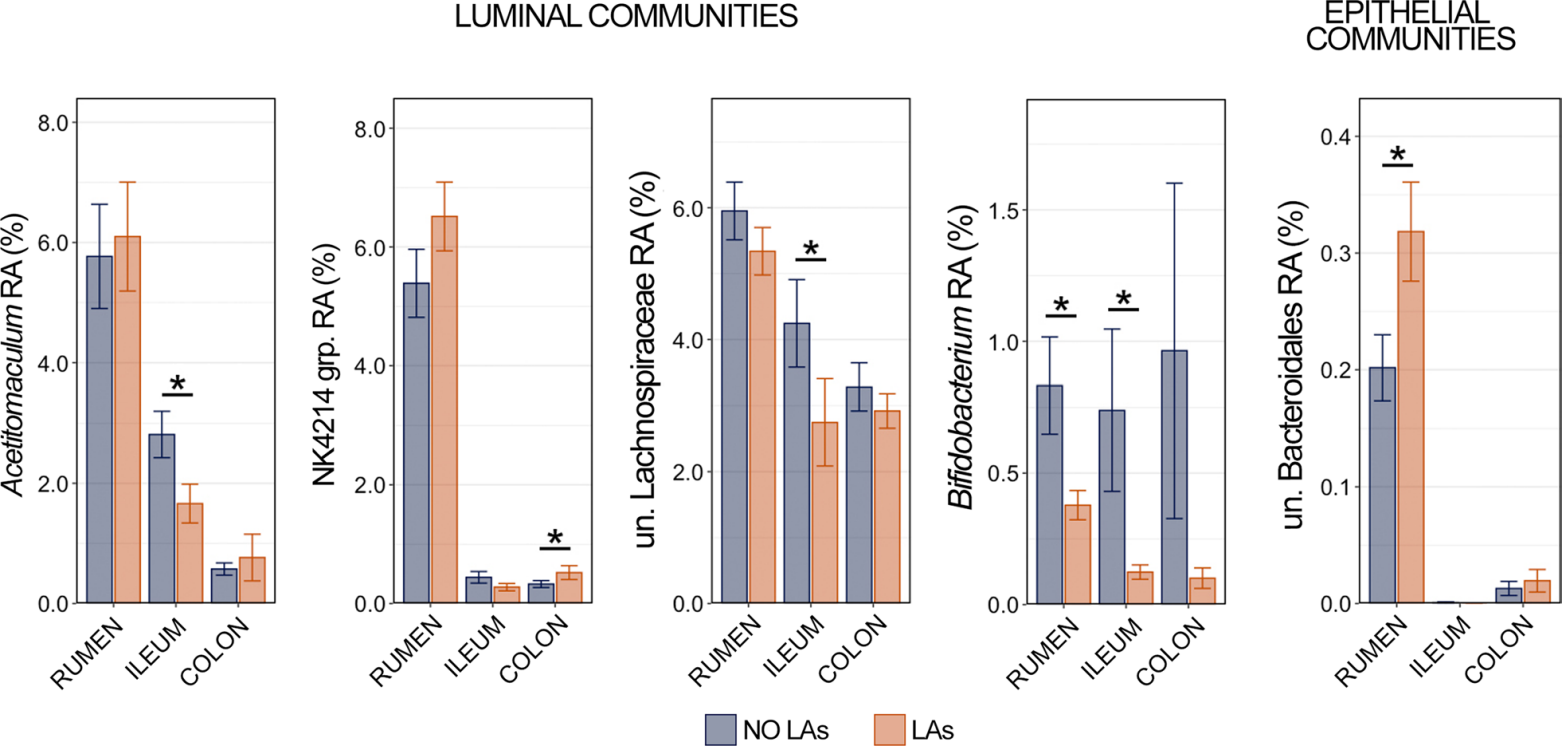
Bar plots demonstrating the relative abundance of the 5 genera identified as differentially abundant within all three GIT locations between animals with and without liver abscesses (LAs) using linear discriminant analysis effect size. Differentially abundant genera in luminal communities (*Acetitomaculum*, NKA4214 group, unclassified Lachnospiraceae, and *Bifidobacterium*) are shown on the left, while the genus-level differentially abundant taxon within epithelial communities (unclassified Bacteroidales) is shown on the left. Significant differences in relative abundance between animals with and without liver abscesses are illustrated by an asterisk (pairwise Wilcoxon rank-sum, n = 15–20, *p* < 0.05)

Linear discriminant analysis effect size identified five genera that were differentially abundant between animals with and without LAs in rumen, ileum, or colon communities (Fig. 9). Four genera (*Acetitomaculum,* NK4A214 group, unclassified Lachnospiraceae, and *Bifidobacterium*) were differentially abundant within luminal communities, and only unclassified Bacteroidales was differentially abundant in epithelial communities. *Acetitomaculum* and unclassified Lachnospiraceae were more abundant in the lumen of the ileum from animals without LAs, while the NK4A214 group was more in luminal communities from the colon of animals with LAs. *Bifidobacterium* was more abundant within luminal communities in the rumen and ileum of animals without LAs compared to those with LAs (Fig. 9; Kruskal–Wallis analysis of variance, n = 15–20, *p* < 0.05). Unclassified Bacteroidales was more abundant within the epithelial communities of the rumen from animals with LAs, and represented the only differentially abundant epithelial taxa (Fig. 9; Kruskal–Wallis analysis of variance, n = 15–20, *p* < 0.05).

**Fig. 10:**
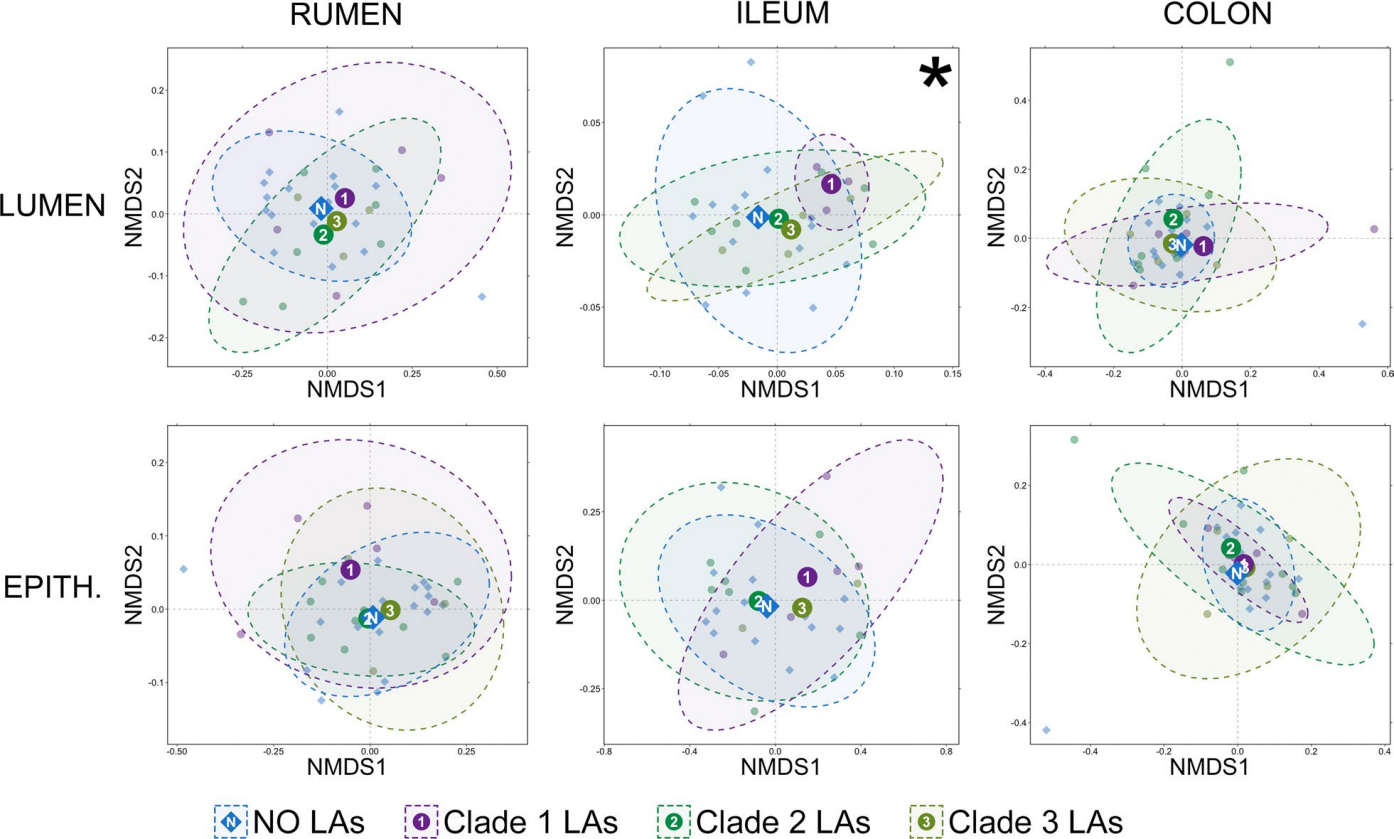
Non-metric multidimensional scaling (NMDS) of generalized UniFrac distances illustrating differences in overall composition of luminal and epithelial microbial communities in the rumen, small intestine, and and large intestine. The NMDS demonstrates clustering of 16S rRNA gene sequences from animals that had no liver abscesses (blue), had liver abscesses classified as clade 1 (Fusobacteriaceae dominant; purple), clade 2 (high Bacteroidaceae; sea green), or clade 3 (high diversity; olive green). The large opaque points represent the centroid for each liver abscess community type, while the smaller and more transparent points represent the individual animals within each group. Dashed lines and shaded areas represent 90% confidence intervals for each liver abscess community type. There was a significant difference in small intestine luminal communities between animals with no liver abscesses and animals with clade 1 (Fusobacteriaceae dominated) abscesses (PERMANOVA, n = 3–15, *p* < 0.05)

Based on generalized UniFrac distances, significant differences in the community composition existed in the lumen of the ileum between animals that did not have liver abscesses and those that had clade 1 (high Fusobacteria) abscesses (Fig. 10; pairwise PERMANOVA, n = 4–10, *p* < 0.05). While a significant PERMDISP suggests that unequal dispersions of variance (pairwise PERMDISP, n = 4–10, *p* < 0.05) is at least partially responsible for the significant PERMANOVA, the distinct clustering of these two groups (Fig. 10) suggests that community composition likely also differed between animals without liver abscesses and those with clade 1 abscess communities. There were no significant differences in community composition between animals without liver abscesses and those with any of the different liver abscess community types within luminal or epithelial communities in the rumen or large intestine (Fig. 10; pairwise PERMANOVA, n = 4–10, *p* > 0.05).

## Discussion

Tylosin supplementation had no impact on the microbial flora of LAs but did change the composition of microbial communities in the rumen and ileum, largely through influencing the predominance of members of the class Clostridia. In the colon, tylosin impacted a few genera but did not shift overall community composition. Interestingly, members of *Turicibacter*—a genus of butyrate producing bacteria that has been shown to decrease in abundance during colitis challenge models in rodents [20]—was more abundant in the colon of animals that received tylosin, suggesting a potential protective effect of tylosin in the hindgut. The lack of impact on LA taxa was expected based on the growing body of literature that has demonstrated this finding [9, 11–13]. Previous work demonstrated that at the phylum level there was no difference in community composition in the GIT following tylosin, but the same study identified changes in the abundances of *Ruminococcus*, Erysipelotrichaceae, and Lachnospiraceae [14]. Interestingly, two of the three taxa they identified (*Ruminococcus* and Lachnospiraceae) were members of the class Clostridia and were also identified as differentially abundant in our study. However, we observed an opposite trend in the abundances of Lachnospiraceae and Erysipelotrichaceae, where these families and the genera comprising them were largely more abundant following tylosin supplementation. A possible explanation for the contrasting results is that the previous study utilized an enzyme-based DNA enrichment method [21] to deplete host DNA before performing shotgun metagenomic sequencing [14], while we did not perform a DNA enrichment and used amplicon-based 16S rRNA sequencing. While both studies are internally sound, comparisons between the two are difficult as it has been demonstrated that differences in techniques like extraction, DNA enrichment, library preparation and sequencing method can impact results [22–25]. Regardless, members of Clostridia appear to be the taxa in the bovine GIT most impacted by the supplementation of tylosin. More specifically members of the family Lachnospiraceae and genus *Ruminococcus*, both core members of the rumen microbiome [26, 27] and abundant members of the bovine hindgut as well, were impacted by the supplementation of tylosin in the rumen and ileum, and to a lesser extent the colon. As a result of its mode of action, tylosin’s activity is largely limited to Gram-positive organisms [28]. Further, members of Clostridia have been widely reported as susceptible to tylosin within the GITs of multiple species in both culture-dependent and culture-independent studies. [29–32]. Due to this, we suggest that alternative treatment methods targeting Clostridia may be worth investigating.

Here, we add to the concept that microbial taxa other than members of Fusobacteria dominate the communities within a significant portion of LAs, which we initially demonstrated in a previous trial [9] and is substantiated by other research [13]. Importantly, multiple LA communities from one animal were highly similar to each other, suggesting they may all be seeded from a localized location in the GIT. For example, from any one given animal, multiple LAs were either Fusobacteria-dominated or Bacteroidetes-dominated. Based on the classification system proposed by Pinnell et al. [9], 12 of the 20 animals in this study (60%) had Bacteroidetes-dominated LAs, which is considerably higher than the proportions of Bacteroidetes-dominated LAs from the two previous studies (23% and 35%) that explicitly reported that proportion [9, 13]. However, our group has reported similarly higher proportions of Bacteroidetes-dominated LAs in a review showing results from two unpublished trials [10]. In this study, high-Bacteroidetes LAs were actually split into two major clades, with one (clade 2 in this sudy) largely comprised of just *Bacteroides* and the other (clade 3 in this study) comprised of *Bacteroides, Porphyromonas*, and taxa from other phyla (i.e., *Trueperella*). Given the apparent trial to trial variation in the proportions of different LA community types, there are likely external factors (i.e., geography, diet, environmental conditions) influencing LA community structure. It is well established that these external factors impact the taxonomy and function of GIT microbial communities in ruminants [33–36], but further work that includes information about the environmental conditions throughout an animal’s life is needed to associate them with different LA microbial taxa.

Bacterial translocation from the lumen of the GIT into the portal vein provides a means for GIT microbiota to seed the liver [3], and the established model for LA pathogenesis focuses on increased gut permeability in the rumen as a result of acidosis [37]. Recent work by our group provided preliminary evidence that non-Fusobacterium dominated LAs may be seeded from more distal portions of the GIT. Here, using samples collected from the same animals with LAs, we demonstrated that the prevalent taxa with non-Fusobacterium LAs (clade 2 and clade 3 in this study) were far more abundant in the ileum and colon then the rumen. We acknowledge the limitations of investigating prevalent LA taxa in the GIT, namely that some prevalent taxa (i.e., *Fusobacterium*, *Porphyromonas*) are in extremely low abundance throughout the GIT of cattle. However, the predominance of *Bacteroides* in the hindgut and its near absence in the rumen of cattle has been demonstrated in numerous studies [38–41], and here we show that in the GIT of the same cattle with LAs *Bacteroides* is virtually absent from the rumen, suggesting that these LAs with abundant *Bacteroides* may originate in the hindgut.

Alterations in the GIT microbiota (dysbiosis) are ubiquitously found to be associated with gut barrier dysfunction (GBD)-related liver disease in humans [42, 43], though which underlies the other is a debate [44]. Here, we identified five genera that were differentially abundant in the GIT of cattle with or without LAs. One of the genera more abundant in the rumen and ileum of animals without LAs was *Bifidobacterium*, which is widely considered to exhibit protective effects against GBD in humans and are commonly identified as discriminant of healthy GIT communities [45–47]. Due to its protective associations, *Bifidobacterium* spp. are commonly used as probiotics in efforts to reduce GBD-related disease in humans [48, 49]. The inclusion of *Bifidobacterium* here as the only taxa differentially abundant in two GIT locations from cattle without LAs suggest it may play a similarly protective role again GBD in cattle. As is the case with any 16S rRNA based investigation we must acknowledge the limitations of such work, specifically that we are limited to relative abundance and lack absolute abundance values. Further work investigating the role of GBD throughout the GIT of cattle should incorporate techniques to provide absolute quantification values for important taxa of interest and include complimentary assays to quantify gut inflammatory responses.

## Conclusions

Dietary inclusion of tylosin (the most common intervention used to prevent and reduce LA occurrence) impacted the microbial communities of the rumen, small intestine, and large intestine of cattle, with the largest impact being observed in the rumen. Furthermore, the first analysis of cattle GIT and liver abscesses microbial communities from the same individual animals suggested that liver abscess communities may be seeded from the hindgut of cattle. The results presented here present the first direct evidence that non-*Fusobacterium* dominated liver abscess communities may in part be seeded from the hindgut of cattle and that multiple abscesses within an individual animal may arise from the same source in the GIT.

## Methods

### Study overview

The study reported here was conducted using a subset of animals enrolled in a blinded, randomized controlled trial evaluating the efficacy of different tylosin supplementation strategies for prevention of LAs using methods meeting Good Clinical Research Practice (GCP) expectations. The subset of cattle used in this investigation were part of the animals randomly assigned to two treatment groups at the time they arrived at the feedlot. The two treatment groups were housed separately in different pens, and the finishing diet of one group was supplemented with tylosin phosphate (90 mg/day for each animal), whereas the diet of the other group was not supplemented with tylosin. Except for this difference, the management protocols were identical for all cattle. The clinical trial methods and management of study animals were reviewed and approved by the West Texas A&M Institutional Animal Care and Use Committee (approved protocol # 2021.08.002) prior to initiation of the study. When cattle reached target harvest weight and body condition, both study groups were harvested on the same day at a federally-inspected commercial abattoir. During carcass evisceration, 10 individual cattle with LAs and 10 without LAs were selected from both treatment groups for inclusion in this study (n = 40 total cattle, 20 with LAs and 20 without LAs). Purulent material from up to 5 LAs were collected from cattle with LAs, and samples of luminal contents and epithelial surface were collected from rumen, ileum, and colon from all animals. All samples were analyzed using 16S rRNA gene sequencing to characterize the microbial communities, and statistical analyses were performed to address the study objectives. Study personnel involved in cattle management, sampling, analysis, and data interpretation were blinded to treatment group assignments throughout the study, from enrollment until after data analyses were completed.

### Study subjects and management

Cattle enrolled in the clinical trial were purchased through commercial marketing sources, targeting English and continental beef breed *Bos taurus* steers (and crossbred animals) with minimal *Bos indicus* influence that weighed 800–950 lb at the time of purchase and were previously raised without substantial grain supplementation. After shipping to a very large commercial feedlot located in Colorado, individual cattle were randomly assigned to one of four study groups, with cattle housed separately by group. The subset of cattle selected for this study were enrolled at the same time, including animals from 10 different marketing groups that were randomly assigned by computer algorithm to pens that housed ~ 250 animals each. At the time of arrival, each animal received a unique identification tag, weighed individually, and administered standard preventive treatments including anthelmintics (ivermectin and fenbendazole), vaccines targeting respiratory disease agents and clostridial bacteria (Bovishield® IBR/BVD Gold and UltraChoice® 7), and a hormone implant to optimize weight gain and feed efficiency (200 mg trenbolone acetate and 40 mg estradiol—Revalor®-XS). Care and management of animals was overseen by the cattle managers and a veterinarian. Additionally, trained animal healthcare personnel visually evaluated every animal daily while riding through pens on horseback. Animals that were potentially ill or injured were moved to the dedicated veterinary facility for examination, and animals meeting established case definitions were treated under the supervision of the feedlot veterinarian using standardized treatment protocols.

### Feeding management and dietary interventions for LAs

The feedlot facilities were constructed to meet or exceed industry standards for beef feedlots, including pens with 130–160 sq-ft of housing space per animal and 7–9 in of linear space of feed bunk per animal. Clean drinking water was provided ad libitum. Diets fed to animals were formulated by animal nutritionists to meet or exceed National Academies of Science nutritional requirements for growing beef cattle, [50], and rations were prepared in accordance with best-practice standards and FDA regulations pertaining to medicated feed articles. Rations were mixed in an FDA-approved Medicated Feed Mill (FDA License number 501–528) that was licensed to manufacture Type C medicated feed using Type A and Type B mediated articles, such as tylosin phosphate.

All cattle were fed the same diets, except for differences in supplementation with the test article, tylosin phosphate, according to treatment group assignments. At the time of arrival, cattle were fed starting ration that was high in forage (hay and silage) until enrollment for ~ 2 weeks, at which time a series of rations were introduced over ~ 21d, which gradually increased the amount of starch so as to prevent the occurrence of acute ruminal acidosis. At that time, the finishing rations for the treatment groups was introduced, including supplementation with tylosin phosphate at 90 mg/animal/day that treatment group. Finishing rations consisted of steam-flaked corn as the grain constituent, corn silage as roughage, dry distillers grains as a protein supplement, whey permeate, a fat source, and supplement containing mineral and vitamins. Monensin (an ionophore) was included in all diets (30 mg/kg of dietary dry matter) to improve feed efficiency, per industry standards. Feed was delivered twice daily in specialized feed trucks containing onboard scales and computers to ensure accurate delivery of appropriate amounts of feed, and feed consumption was adjusted at least biweekly to ensure appropriate supplementation of tylosin phosphate to meet the intended dosing (90 mg/hd/day). At 33d prior to harvest, ractopamine hydrochloride (Optaflexx®, 280 mg/animal/day) was supplemented in all diets to improve rate of weight gain, feed efficiency, and carcass leanness; this supplementation was removed from rations 2d prior to slaughter, per FDA regulations. It should be noted that monensin and ractopamine supplementation have not been demonstrated to be associated with significant changes in the occurrence of LAs. Nutrient composition of rations were analyzed throughout the study to indirectly assess and refine delivery of the targeted amount of tylosin phosphate in final mixed rations by comparison to the target composition of the ration formula.

Cattle were on feed for 185 days prior harvest, and their average daily gain in body weight over this period was 3.0 lbs per day. The average live body weight of cattle at harvest was 1464 lbs for the untreated pen and 1468 lbs for the pen treated with tylosin. At harvest, the average hot carcass weight (standard deviation) for the two pens was 931 lbs (74 lbs) and 928 lbs (73 lbs), respectively.

### Euthanasia of cattle and sample collection

Study cattle were euthanized and harvested at a USDA–FSIS inspected commercial abattoir using approved and supervised methods. All animals from each study group were processed separately, and during carcass evisceration, a study investigator (PSM) selected by convenience 10 individual cattle with LAs and 10 without LAs from both treatment groups for inclusion in this study (n = 40 total cattle, 20 with LAs and 20 without LAs). Among those with LAs, cattle with severe abscessation (A or A + based on the Elanco scoring system) were preferentially selected for inclusion. A study ID number was assigned to each animal at the time of selection and used to identify all samples collected from the same individuals. Blocks of liver tissue containing abscesses were excised, placed in sterile bags, and refrigerated on ice. The rumen was opened onsite at the slaughter facility, and samples of luminal content and mucosal surface swabs (1.3 cm diameter rayon tipped swabs, Puritan®), were collected aseptically from the ventral rumen sac, placed in sterile tubes, and refrigerated on ice. Approximately 20 cm proximal to the cecum, sections of ileum (15–20 cm in length) were tied off and excised, placed in sterile bags, and refrigerated on ice. Similarly, 15–20 cm of the distal colon loop was tied off, excised, placed in sterile bags, and refrigerated on ice. All samples were transported to laboratory facilities at Colorado State University for further processing within 6 h of collection.

Prior to collecting samples for sequencing, external surfaces of tissues were flame sterilized after spraying with 70% ethanol. Tissues were opened using sterile disposable scalpels, and LA purulent material and intestinal contents were collected aseptically using sterile disposable spatulas and placed in sterile cryotubes. A total of 53 abscesses, up to 5 per steer, were collected from the livers of the 20 cattle with LAs. A large (1.3 cm diameter) rayon tipped swab was used to aseptically sample the epithelial surfaces of the ileum and colon and placed in sterile tubes. Epithelial surfaces were not rinsed prior to swabbing to reduce contamination potential, but this does mean there was potential for luminal community members to be present. However, given significant differences in community composition between luminal and epithelial communities in the rumen (see below) we don’t believe this has impact our conclusions. All samples were frozen at − 80 °C after collection until further processed for sequencing.

### DNA isolation, 16S rRNA gene library preparation and sequencing

DNA was isolated from all samples using the QIAamp PowerFecal Pro DNA kit and a QIAcube Connect (Qiagen, Hilden, Germany) automated isolation system for nucleic acid recovery according to the manufacturer’s instructions. Following isolation, DNA was quantified (ng·μL-1) using a Qubit Flex fluorometer (ThermoFisher, Waltham, MA). An extraction blank was included for each sample type (i.e., LA, lumen and epithelium from the rumen, small intestine, and large intestine) that were included in subsequent library preparation steps and sequencing.

The V3-V4 region of the 16S rRNA gene was amplified using the 341f/785r primer pair [51] and 400 ng of template DNA. Amplification conditions were 98 °C for 3 min, followed by 18 cycles of 98 °C for 30 s, 55 °C for 30 s, and 72 °C for one minute. Final elongation occurred at 72 °C for 5 min. Amplicons were then purified using beads (AMPure XP beads, Beckman-Coulter, Pasadena, CA) and sequencing libraries were prepared following the Illumina protocol (Illumina, San Diego, CA). Libraries were purified using AMPure XP beads and pooled in equal proportions based on molarities. To increase the number of sequences per sample, the resulting amplicon library pool was sequenced in two separate runs on an Illumina MiSeq instrument (Illumina, San Diego, CA, USA) using 2 × 250 base pair (bp) paired-end chemistry at the Texas A&M Institute for Genome Sciences and Society sequencing core. Each plate of PCR reactions included a no-template negative control (NTC), which consisted of an equal volume of nuclease-free sterile water as template. These controls were included in the preparation of sequencing libraries. Sequencing of extraction blanks and NTCs did not yield product and therefore they were not included in further downstream analysis.

### Bioinformatics

Demultiplexed 16S rRNA gene sequence reads were imported into QIIME2 version 2022.2 [52]. Because its error correction method performs better on individual sequencing runs, amplicon sequence variants (ASVs) were generated for each of the two sequencing runs separately using DADA2 [53], which also filtered reads for quality, removed chimeric sequences, and merged overlapping paired-end reads. Forward reads were trimmed at 17 bp, and reverse reads were trimmed at 21 bp for both runs, while both forward reads were truncated at 250 bp and reverse reads were truncated at 248 bp for both runs. The resulting ASV tables and representative sequences were then merged using the ‘qiime feature-table merge’ and ‘qiime feature-table merge-seqs’ functions, respectively. The merging was performed by using the ‘sum’ method, which combined individual ASV counts from the two sequencing runs on the same pooled sample. Taxonomy was assigned using a Naïve Bayes classifier trained on the SILVA 138.1 SSU NR 99 database [54], where sequences had been trimmed to only include those base pairs from the V3-V4 region bound by the 341f/785r primer pair. Reads mapping to chloroplast and mitochondrial sequences were removed from the ASV table and representative sequences, and a mid-point rooted phylogenetic tree was generated using ‘qiime alignment mafft’, ‘qiime alignment mask’, and ‘qiime phylogeny fasttree’ under default settings. The ASV table, representative sequences, and mid-point rooted tree were then imported into phyloseq [55] using the ‘import_biom’ function. Metadata was imported using the ‘import_qiime_sample_data’ and merged with the ASV table, representative sequences, and tree into a phyloseq object. Samples with less than 10,000 ASVs (n = 9) were discarded and omitted from downstream analysis. Remaining samples (n = 256) had a range of 17,120 ASVs to 119,736 ASVs per sample and an average of 51,093 ASVs per sample. There was no significant difference in the number of ASVs per sample between animals with or without LAs in samples collected from the rumen, small intestine, or large intestine (Kruskal–Wallis analysis of variance, n = 30–38, *p* > 0.05), and no difference in the number of ASVs per sample between liver abscesses classified as high Fusobacteria or high Bacteroidetes (Kruskal–Wallis analysis of variance, n = 20–32, *p* > 0.05). Similarly, there was no significant difference in the number of ASVs per samples between animals receiving tylosin and those that did not (Kruskal–Wallis analysis of variance, n = 114–142, *p* > 0.05). Over 91% of all ASVs were classified at the level of genus and > 99% of all ASVs were classified at the ranks of family, order, class, and phylum from all four body sites sampled (i.e., LAs, rumen, small intestine, large intestine). Luminal and epithelial communities were significantly different from each other in the rumen (PERMANOVA, n = 15–20, *p* < 0.05), and as such all luminal and epithelial communities were analyzed separately.

Richness (observed ASVs) and Faith’s phylogenetic distance (FPD) were calculated for all remaining samples (n = 256) with phyloseq and the ‘estimate_pd’ function from the btools package. ASV counts were then normalized using cumulative sum scaling [56] and beta-diversity was analyzed using generalized UniFrac distances [57, 58]. From these distances, non-metric multidimensional scaling (NMDS) was performed and plotted, and permutational multivariate analysis of variance (PERMANOVA) was used to test for significant differences in community structure using the vegan [59] and pairwiseAdonis [60] packages. To ensure significant differences were not the result of unequal dispersions of variance between groups, permutational analysis of dispersion (PERMDISP) were conducted for all significant PERMANOVA outcomes using vegan. Additionally, hierarchal clustering was performed on generalized UniFrac distances using Ward’s agglomeration method [61] and the ‘hclust’ function. Dendrograms were created from the hierarchal clustering results using the ‘ggdendro’ package. Further, the relative abundances of normalized ASVs within each sample were calculated and plotted using phyloseq. To be considered prevalent, microbial taxa had to be present in at least 40% of samples from one of the three major clades of LAs and be in an average relative abundance of greater than 0.01% across those samples.

Differentially abundant genera were identified in the rumen, ileum, and colon between animals with and without LAs using linear discriminant analysis effect size (LEfSe) performed with the online LEfSe tool on the Galaxy server under default settings. In an effort to avoid potential false positives, a more conservative pairwise Wilcoxon rank-sum test was used to statistically test differences genera identified as differentially abundant with LEfSe between animals with and without LAs.

### Statistical analysis

Unless specified otherwise, R version 4.2.1 was used for statistical analysis of data. Kruskal–Wallis analysis of variance (for comparisons between 2 variables) or Pairwise Wilcoxon rank-sum tests were performed with a Benjamini–Hochberg correction for multiple comparisons (for comparisons between more than 2 variables). Differences in beta-diversity were tested using pairwise PERMANOVA with a Benjamini–Hochberg correction for multiple comparisons and 9999 permutations. Additionally, pairwise PERMDISPs were carried out for all significant PERMANOVA outcomes using 9999 permutations to test for differences in the variability of dispersions.

### Supplementary Information

**Additional file 1**. **Table S1** Relative abundances of taxonomic families comprising more than 1% of the overall community across all rumen samples. The mean relative abundance and standard error of the mean are displayed for each family from luminal and epithelial communities of the rumen from animals that received tylosin supplementation and those that did not. Significant p-values are bold (Kruskal-Wallis analysis of variance).

**Additional file 2**. **Table S2** Taxonomic information for the 20 genera differentially abundant across all sample sites between animals receiving tylosin supplementation and those that did not.

**Additional file 3**. **Table S3** Relative abundances of taxonomic families comprising more than 1% of the overall community across all small intestine samples. The mean relative abundance and standard error of the mean are displayed for each family from luminal and epithelial communities of the small intestine from animals that received tylosin supplementation and those that did not. Significant p-values are bolded (Kruskal-Wallis analysis of variance).

**Additional file 4**. **Table S4** Relative abundances of taxonomic families comprising more than 1% of the overall community across all large intestine samples. The mean relative abundance and standard error of the mean are displayed for each family from luminal and epithelial communities of the small intestine from animals that received tylosin supplementation and those that did not. Significant p-values are bolded (Kruskal-Wallis analysis of variance).

**Additional file 5**. **Fig. S1** Non-metric multidimensional scaling (NMDS) of generalized UniFrac distances illustrates differences in overall microbial community structure of liver abscesses between treatment groups. The NMDS demonstrates clustering of 16S rRNA gene sequences from animals that received tylosin (purple) and those that did not (gold). There were no significant differences in overall community composition (PERMANOVA, *p* > 0.05, n = 10). The bar plot demonstrates the mean relative abundance of all genera within liver abscesses that comprised at least 0.5% of the overall microbial community between animals that received tylosin supplementation and those that did not. Error bars represent the standard error of the mean. There were no significant differences detected (Kruskal–Wallis analysis of variance; n = 10; *p* < 0.05). To limit the effect of some animals having multiple (i.e., up to 5) abscess communities, values were based on normalized ASV counts generated by averaging counts from each liver abscess within an individual animal.

**Additional file 6**. **Fig. S2** Bar plots demonstrating the relative abundances of taxonomic families within purulent material from individual abscesses (n = 47) from the 14 animals that contained multiple liver abscesses were collected. The eight most abundant families are displayed in the legend.

**Additional file 7**. **Fig. S3** Boxplots demonstrating the relative abundance of *Bacteroides* across luminal and epithelial microbial communities in the rumen, ileum, and colon. Significant differences are illustrated by different letters (pairwise Wilcoxon rank-sum, n = 59–70, *p* < 0.05).

## Data Availability

All sequence reads were made available through BioProject PRJNA904691 at the NCBI’s Sequence Read Archive. The code and instructions for the bioinformatic and statistical analyses can be found at this GitHub repository: https://github.com/ljpinnell/Bovine_LiverAbscess_GIT.
