# Supplementary material for: Establishing the link between microbial communities in bovine liver abscesses and the gastrointestinal tract
Source: Anim Microbiome. 2023 Nov 20;5:58. doi: 10.1186/s42523-023-00278-0 (PMC10662489; doi:10.1186/s42523-023-00278-0)
Supplement: Supplementary file 1 — Additional file 1. Table S1 Relative abundances of taxonomic families comprising more than 1% of the overall community across all rumen samples. The mean relative abundance and standard error of the mean are displayed for each family from luminal and epithelial communities of the rumen from animals that received tylosin supplementation and those that did not. Significant p-values are bold (Kruskal-Wallis analysis of variance). [file 42523_2023_278_MOESM1_ESM.docx]

**Table S1.** Relative abundances of taxonomic families comprising more than 1% of the overall community across all rumen samples. The mean relative abundance and standard error of the mean are displayed for each family from luminal and epithelial communities of the rumen from animals that received tylosin supplementation and those that did not. Significant p-values are bold (Kruskal-Wallis analysis of variance).

| **LUMEN** | **NO TYLOSIN (n = 15)** | | | | **TYLOSIN (n = 19)** | | |  |
| --- | --- | --- | --- | --- | --- | --- | --- | --- |
|  | **Mean** | **SEM** | | | **Mean** | | **SEM** | **p-val.** |
| Lachnospiraceae | 27.14 | 2.516 | | | 35.39 | | 1.984 | **0.019** |
| Prevotellaceae | 17.94 | 1.356 | | | 15.37 | | 1.506 | 0.193 |
| Atopobiaceae | 8.85 | 1.128 | | | 9.96 | | 0.611 | 0.201 |
| Oscillospiraceae | 7.80 | 0.687 | | | 6.20 | | 0.650 | 0.074 |
| Methanobacteriaceae | 5.02 | 0.539 | | | 4.18 | | 0.351 | 0.218 |
| Anaerovoracaceae | 3.88 | 0.411 | | | 2.86 | | 0.335 | 0.069 |
| Ruminococcaceae | 3.12 | 0.642 | | | 3.20 | | 1.021 | 0.245 |
| Muribaculaceae | 3.22 | 0.767 | | | 1.93 | | 0.519 | 0.107 |
| Clostridia UCG-014 | 1.56 | 0.238 | | | 2.88 | | 0.313 | **0.003** |
| Erysipelotrichaceae | 2.01 | 0.206 | | | 2.43 | | 0.209 | 0.245 |
| Acidaminococcaceae | 1.45 | 0.168 | | | 1.52 | | 0.127 | 0.499 |
| Rikenellaceae | 1.81 | 0.325 | | | 1.04 | | 0.168 | 0.252 |
| Selenomonadaceae | 1.26 | 0.375 | | | 1.44 | | 0.279 | 0.396 |
| Bacteroidales RF16 grp. | 1.49 | 0.386 | | | 0.71 | | 0.190 | 0.127 |
| Eub. coprostanoligenes grp. | 1.14 | 0.207 | | | 0.95 | | 0.127 | 0.377 |
|  |  | |  |  | |  | |  |
| **EPITHELIUM** | **NO TYLOSIN (n = 17)** | | | | **TYLOSIN (n = 20)** | | |  |
|  | **Mean** | **SEM** | | | **Mean** | | **SEM** | **p-val.** |
| Prevotellaceae | 33.28 | 1.011 | | | 33.40 | | 0.766 | 0.976 |
| Lachnospiraceae | 20.16 | 1.088 | | | 20.74 | | 0.746 | 0.784 |
| Succinivibrionaceae | 6.34 | 1.252 | | | 14.29 | | 1.798 | **0.005** |
| Selenomonadaceae | 5.02 | 0.495 | | | 4.25 | | 0.233 | 0.161 |
| Veillonellaceae | 2.85 | 0.309 | | | 3.64 | | 0.241 | **0.041** |
| Atopobiaceae | 2.90 | 0.312 | | | 2.87 | | 0.192 | 0.855 |
| Oscillospiraceae | 3.43 | 0.430 | | | 2.22 | | 0.232 | **0.021** |
| Ruminococcaceae | 3.01 | 0.514 | | | 2.33 | | 0.693 | **0.048** |
| Muribaculaceae | 2.70 | 0.498 | | | 2.11 | | 0.523 | 0.273 |
| Anaerovoracaceae | 2.50 | 0.359 | | | 1.71 | | 0.166 | 0.051 |
| Acidaminococcaceae | 1.76 | 0.228 | | | 1.88 | | 0.167 | 0.273 |
| Rikenellaceae | 2.33 | 0.393 | | | 1.31 | | 0.177 | **0.035** |
| Methanobacteriaceae | 1.73 | 0.196 | | | 1.37 | | 0.129 | 0.190 |
| Clostridia UCG-014 | 0.79 | 0.094 | | | 1.23 | | 0.122 | **0.015** |

Abbreviations: Eub., Eubacterium; val., value; SEM, standard error of the mean
