# Supplementary material for: Establishing the link between microbial communities in bovine liver abscesses and the gastrointestinal tract
Source: Anim Microbiome. 2023 Nov 20;5:58. doi: 10.1186/s42523-023-00278-0 (PMC10662489; doi:10.1186/s42523-023-00278-0)
Supplement: Supplementary file 2 — Additional file 2. Table S2 Taxonomic information for the 20 genera differentially abundant across all sample sites between animals receiving tylosin supplementation and those that did not. [file 42523_2023_278_MOESM2_ESM.docx]

**Table S2.** Taxonomic information for the 20 genera differentially abundant across all sample sites between animals receiving tylosin supplementation and those that did not.

| **Class** | **Order** | **Family** | **Genus** |
| --- | --- | --- | --- |
| Bacilli | Erysipelotrichales | Erysipelatoclostridiaceae | UCG-004 |
| Bacilli | Erysipelotrichales | Erysipelotrichaceae | UCG-009 |
| Bacilli | Erysipelotrichales | Erysipelotrichaceae | *Turicibacter* |
| Bacteroidia | Bacteroidales | F082 | F082 |
| Bacteroidia | Bacteroidales | Prevotellaceae | UCG-001 |
| Bacteroidia | Bacteroidales | Rikenellaceae | RC9 gut group |
| Clostridia | Clostridia_UCG-014 | Clostridia_UCG-014 | Clostridia UCG-014 |
| Clostridia | Lachnospirales | Lachnospiraceae | Ruminococcus gauvreauii group |
| Clostridia | Lachnospirales | Lachnospiraceae | *Shuttleworthia* |
| Clostridia | Lachnospirales | Lachnospiraceae | Un. Lachnospiraceae |
| Clostridia | Oscillospirales | Eubacterium coprostanoligenes group | Eubacterium coprostanoligenes group |
| Clostridia | Oscillospirales | Oscillospiraceae | NK4A214_group |
| Clostridia | Oscillospirales | Ruminococcaceae | *Ruminococcus* |
| Clostridia | Oscillospirales | Ruminococcaceae | un. Ruminococcaceae |
| Clostridia | Peptostreptococcales-Tissierellales | Anaerovoracaceae | Eubacterium nodatum_group |
| Clostridia | Peptostreptococcales-Tissierellales | Anaerovoracaceae | Family XIII AD3011 group |
| Clostridia | Peptostreptococcales-Tissierellales | Anaerovoracaceae | *Mogibacterium* |
| Coriobacteriia | Coriobacteriales | Atopobiaceae | *Olsenella* |
| γ-proteobacteria | Aeromonadales | Succinivibrionaceae | UCG-001 |
| Methanobacteria | Methanobacteriales | Methanobacteriaceae | *Methanobrevibacter* |
