# Supplementary material for: Establishing the link between microbial communities in bovine liver abscesses and the gastrointestinal tract
Source: Anim Microbiome. 2023 Nov 20;5:58. doi: 10.1186/s42523-023-00278-0 (PMC10662489; doi:10.1186/s42523-023-00278-0)
Supplement: Supplementary file 4 — Additional file 4. Table S4 Relative abundances of taxonomic families comprising more than 1% of the overall community across all large intestine samples. The mean relative abundance and standard error of the mean are displayed for each family from luminal and epithelial communities of the small intestine from animals that received tylosin supplementation and those that did not. Significant p-values are bolded (Kruskal-Wallis analysis of variance). [file 42523_2023_278_MOESM4_ESM.docx]

**Table S4.** Relative abundances of taxonomic families comprising more than 1% of the overall community across all large intestine samples. The mean relative abundance and standard error of the mean are displayed for each family from luminal and epithelial communities of the small intestine from animals that received tylosin supplementation and those that did not. Significant p-values are bolded (Kruskal-Wallis analysis of variance).

| **LUMEN** | **NO TYLOSIN (n = 17)** | | **TYLOSIN (n = 19)** | |  |
| --- | --- | --- | --- | --- | --- |
|  | **Mean** | **SEM** | **Mean** | **SEM** | **p-val.** |
| Lachnospiraceae | 15.25 | 3.332 | 13.24 | 1.258 | 0.862 |
| Peptostreptococcaceae | 10.23 | 1.127 | 11.31 | 0.858 | 0.168 |
| Oscillospiraceae | 10.56 | 1.114 | 9.73 | 0.477 | 0.159 |
| Prevotellaceae | 9.99 | 1.863 | 7.21 | 0.897 | 0.199 |
| Bacteroidaceae | 6.31 | 1.114 | 9.50 | 1.087 | 0.103 |
| Clostridiaceae | 6.09 | 1.202 | 7.96 | 0.938 | 0.103 |
| Erysipelotrichaceae | 4.91 | 0.823 | 7.97 | 0.619 | **0.003** |
| Rikenellaceae | 5.09 | 0.980 | 4.92 | 0.501 | 0.962 |
| Muribaculaceae | 3.49 | 0.532 | 3.58 | 0.366 | 0.887 |
| Atopobiaceae | 2.33 | 0.249 | 4.22 | 0.547 | **0.006** |
| Ruminococcaceae | 3.53 | 0.578 | 2.82 | 0.411 | 0.261 |
| Eub. coprostanoligenes grp. | 2.41 | 0.442 | 1.93 | 0.294 | 0.646 |
| Spirochaetaceae | 2.31 | 0.798 | 1.69 | 0.438 | 0.962 |
| Methanobacteriaceae | 1.69 | 0.411 | 2.22 | 0.267 | **0.044** |
| Anaerovoracaceae | 1.68 | 0.476 | 1.01 | 0.084 | **0.030** |
| Oscillospirales UCG-010 | 1.54 | 0.357 | 1.10 | 0.155 | 0.669 |
| Christensenellaceae | 1.27 | 0.290 | 0.90 | 0.145 | 0.516 |
| Monoglobaceae | 1.22 | 0.257 | 0.85 | 0.178 | 0.288 |
|  |  |  |  |  |  |
| **EPITHELIUM** | **NO TYLOSIN (n = 17)** | | **TYLOSIN (n = 18)** | |  |
|  | **Mean** | **SEM** | **Mean** | **SEM** | **p-val.** |
| Lachnospiraceae | 13.65 | 1.684 | 14.16 | 1.111 | 0.468 |
| Prevotellaceae | 12.24 | 1.840 | 9.90 | 1.172 | 0.355 |
| Oscillospiraceae | 10.53 | 1.063 | 10.55 | 0.569 | 0.488 |
| Bacteroidaceae | 7.94 | 1.240 | 10.01 | 1.025 | 0.391 |
| Peptostreptococcaceae | 8.35 | 0.976 | 8.91 | 0.632 | 0.262 |
| Clostridiaceae | 5.83 | 1.203 | 7.00 | 0.814 | 0.080 |
| Erysipelotrichaceae | 4.40 | 0.611 | 6.47 | 0.544 | **0.008** |
| Rikenellaceae | 4.79 | 0.854 | 4.76 | 0.454 | 0.947 |
| Ruminococcaceae | 3.91 | 0.712 | 3.31 | 0.563 | 0.553 |
| Muribaculaceae | 3.30 | 0.423 | 3.37 | 0.309 | 0.869 |
| Atopobiaceae | 1.94 | 0.203 | 3.59 | 0.490 | **0.011** |
| Eub. coprostanoligenes grp. | 2.47 | 0.440 | 2.22 | 0.342 | 0.843 |
| Oscillospirales UCG-010 | 1.91 | 0.396 | 1.68 | 0.206 | 0.921 |
| Spirochaetaceae | 1.98 | 0.562 | 1.61 | 0.410 | 0.817 |
| Anaerovoracaceae | 1.17 | 0.100 | 1.11 | 0.095 | 0.766 |
| Methanobacteriaceae | 0.90 | 0.239 | 1.28 | 0.172 | **0.032** |
| Bifidobacteriaceae | 1.39 | 0.948 | 0.72 | 0.519 | 0.478 |
| Monoglobaceae | 1.13 | 0.236 | 0.93 | 0.166 | 0.869 |
