# Supplementary figures and images for: Establishing the link between microbial communities in bovine liver abscesses and the gastrointestinal tract
Source: Anim Microbiome. 2023 Nov 20;5:58. doi: 10.1186/s42523-023-00278-0 (PMC10662489; doi:10.1186/s42523-023-00278-0)

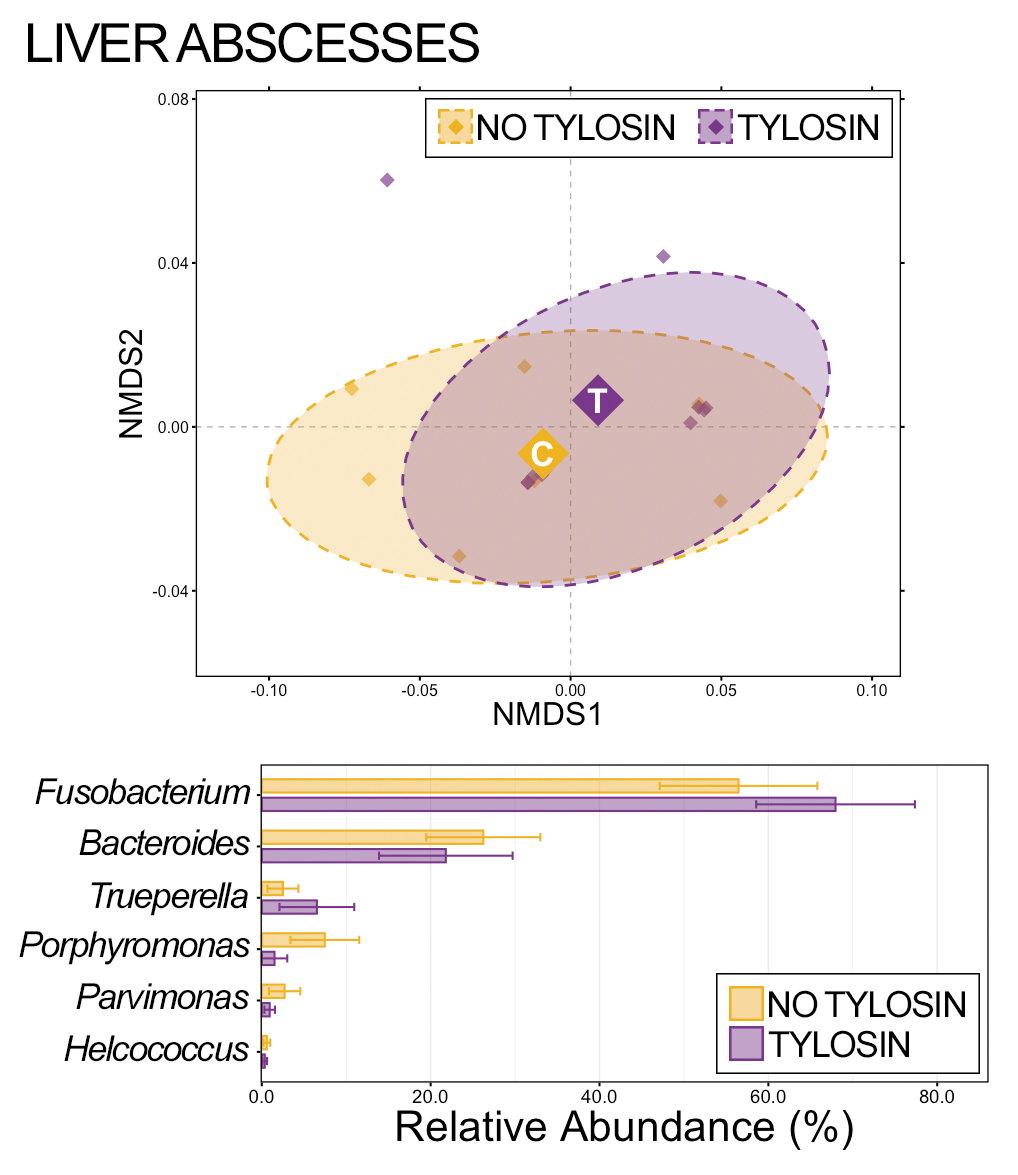

Supplement: Supplementary file 5 — Additional file 5. Fig. S1 Non-metric multidimensional scaling (NMDS) of generalized UniFrac distances illustrates differences in overall microbial community structure of liver abscesses between treatment groups. The NMDS demonstrates clustering of 16S rRNA gene sequences from animals that received tylosin (purple) and those that did not (gold). There were no significant differences in overall community composition (PERMANOVA, p > 0.05, n = 10). The bar plot demonstrates the mean relative abundance of all genera within liver abscesses that comprised at least 0.5% of the overall microbial community between animals that received tylosin supplementation and those that did not. Error bars represent the standard error of the mean. There were no significant differences detected (Kruskal–Wallis analysis of variance; n = 10; p < 0.05). To limit the effect of some animals having multiple (i.e., up to 5) abscess communities, values were based on normalized ASV counts generated by averaging counts from each liver abscess within an individual animal. [file 42523_2023_278_MOESM5_ESM.jpg]

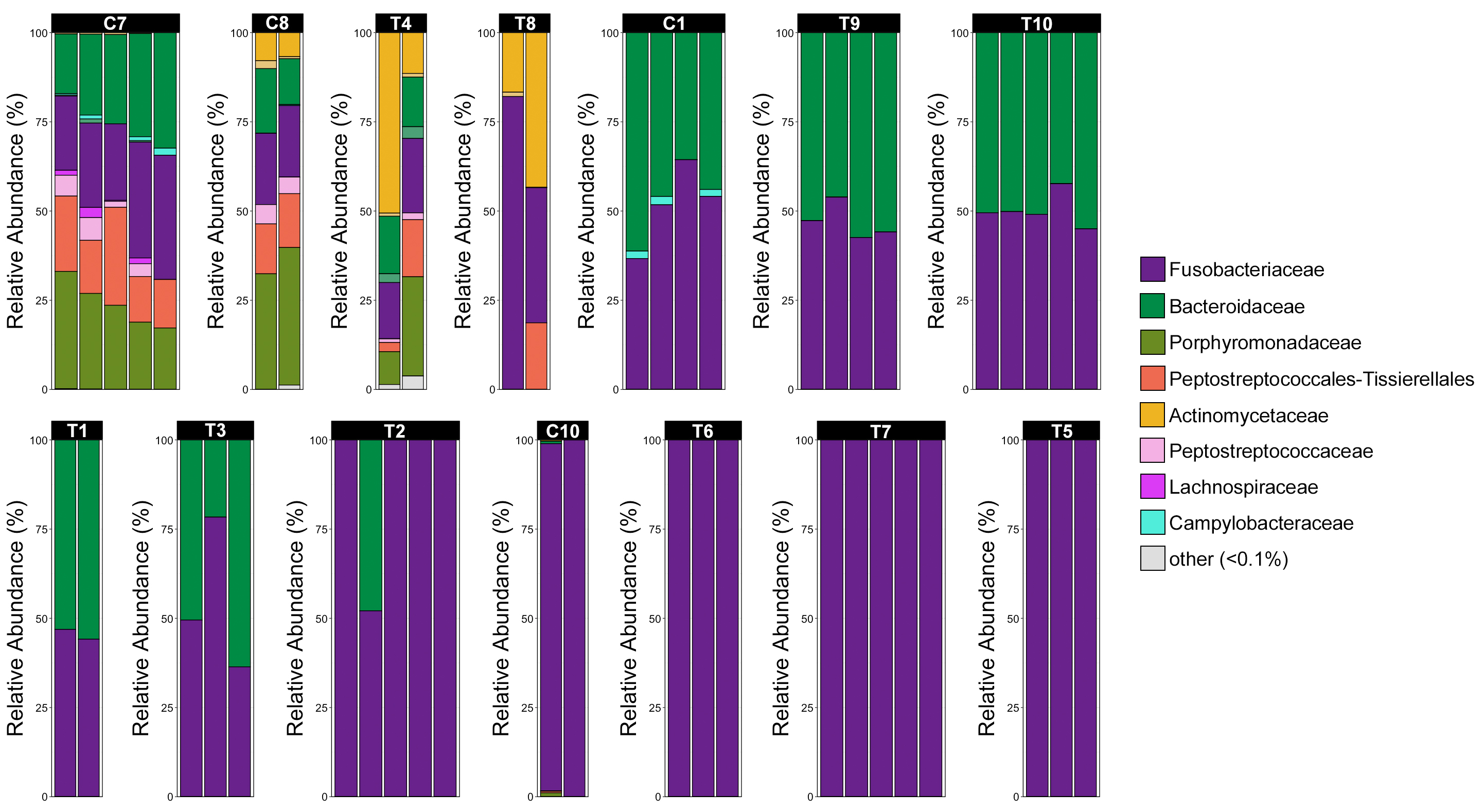

Supplement: Supplementary file 6 — Additional file 6. Fig. S2 Bar plots demonstrating the relative abundances of taxonomic families within purulent material from individual abscesses (n = 47) from the 14 animals that contained multiple liver abscesses were collected. The eight most abundant families are displayed in the legend. [file 42523_2023_278_MOESM6_ESM.jpg]

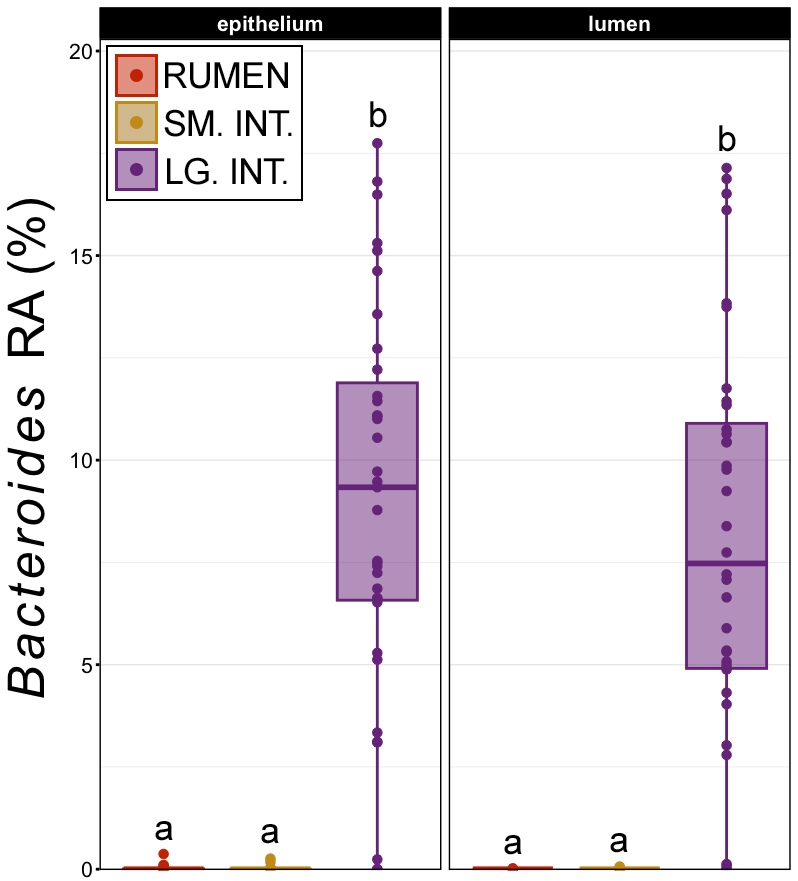

Supplement: Supplementary file 7 — Additional file 7. Fig. S3 Boxplots demonstrating the relative abundance of Bacteroides across luminal and epithelial microbial communities in the rumen, ileum, and colon. Significant differences are illustrated by different letters (pairwise Wilcoxon rank-sum, n = 59–70, p < 0.05). [file 42523_2023_278_MOESM7_ESM.jpg]
